# Effects of visual blur and contrast on spatial and temporal precision in manual interception

**DOI:** 10.1007/s00221-021-06184-8

**Published:** 2021-09-04

**Authors:** Anna Schroeger, J. Walter Tolentino-Castro, Markus Raab, Rouwen Cañal-Bruland

**Affiliations:** 1 Department for the Psychology of Human Movement and Sport, Institute of Sport Science, Friedrich Schiller University Jena, Jena, Germany; 2 Department of Performance Psychology, Institute of Psychology, German Sport University Cologne, Cologne, Germany; 3 School of Applied Sciences, London South Bank University, London, UK

**Keywords:** Temporal precision, Spatial precision, Perception, Action, Interception, Spatiotemporal accuracy

## Abstract

The visual system is said to be especially sensitive towards spatial but lesser so towards temporal information. To test this, in two experiments, we systematically reduced the acuity and contrast of a visual stimulus and examined the impact on spatial and temporal precision (and accuracy) in a manual interception task. In Experiment 1, we blurred a virtual, to-be-intercepted moving circle (ball). Participants were asked to indicate (i.e., finger tap) on a touchscreen where and when the virtual ball crossed a ground line. As a measure of spatial and temporal accuracy and precision, we analyzed the constant and variable errors, respectively. With increasing blur, the spatial and temporal variable error, as well as the spatial constant error increased, while the temporal constant error decreased. Because in the first experiment, blur was potentially confounded with contrast, in Experiment 2, we re-ran the experiment with one difference: instead of blur, we included five levels of contrast matched to the blur levels. We found no systematic effects of contrast. Our findings confirm that blurring vision decreases spatial precision and accuracy and that the effects were not mediated by concomitant changes in contrast. However, blurring vision also affected temporal precision and accuracy, thereby questioning the generalizability of the theoretical predictions to the applied interception task.

**Supplementary Information:**

The online version contains supplementary material available at 10.1007/s00221-021-06184-8.

## Introduction

Visual perception is of utmost importance to guide our actions in daily life. For example, when aiming for a cup of coffee, vision informs us about where to grasp it so as not to tip over the cup and spill the coffee. In dynamic situations, for instance, when catching a fly ball next to spatial also temporal predictions are key (Fischman and Schneider 1985; McBeath 1990; Oudejans et al. 1996; Savelsbergh and Whiting 1988). In such situations, successful actions are characterized by guiding the body or limbs to be in the right place at the right time.

To appropriately plan and control movements, the visual information picked up both in advance and during execution has been shown to make a significant contribution (see also Barany et al. 2020; Lim 2015; Marinovic et al. 2009). Accordingly, if vision is diminished, it has been shown to result in less precise movements (e.g. Zhao and Warren 2017). It was shown that manipulations of visual features such as, for instance, blur (Dehnert et al. 2011; Johnson and Casson 1995), contrast (Chen and Muhamad 2018; Johnson and Casson 1995), colors (Chen and Muhamad 2018), and luminance (Johnson and Casson 1995; Tidbury et al. 2016) impact human perception by diminishing visual acuity (i.e., spatial resolution of the visual system).

Assuming that accurate visual perception is important to guide precise actions (see also Creem and Proffitt 2001), it follows that such reductions of visual acuity should also impact spatiotemporal precision when intercepting moving objects such as when catching fly balls. In fact, Mann et al. (2007) demonstrated that high levels of myopic blur cause reductions in cricket batting performance. Players were asked to bat a ball delivered by a bowling machine under different blur conditions manipulated via differently blurred contact lenses. The highest myopic blur condition (+3 D) resulted in significantly reduced batting performance (percentage of bat-ball contacts) compared to the two smaller refractive conditions (+1 D and +2 D), whilst the other two levels did not differ significantly from normal vision. Hence, the authors concluded that optimal vision is not necessary for optimal interception, but that very high levels of myopic blur can negatively affect batting performance. The authors explain this resilience of cricket players to a wide range of blur with a good compensation of the human perceptual-motor system. They also noticed a maintenance of ‘good’ bat-ball contacts for high levels of blur at the cost of a less aggressive, more conservative strategy resulting in more defensive strokes which might be less efficient in a real cricket game. Similar results were obtained for aiming at stationary targets in golf putting (Bulson et al. 2008) and basketball free throws (Bulson et al. 2015) as well as for interception performance in another cricket study (Mann et al. 2010). Bulson et al. (2008, 2015) provide several explanations of the missing effects for small blur levels: first, blur adaptation may have taken place in their experiments, as it was previously shown that participants adapt to low blur levels already after a few minutes exposure (Wang et al. 2006; Webster et al. 2002). Second, motor learning/motor memory might play an important role. The better a motor task is learned the stronger is the associations between sensory cues and appropriate motor responses and the less sensory input is necessary for movement execution.

Zhao and Warren (2017) recently investigated the effect of visual blur in a virtual interception paradigm. Participants were asked to walk in a virtual open environment towards a moving target, namely a green two-dimensional bar, to intercept it. The target was progressively blurred within each trial until reaching one of five blur levels (including no-blur) or complete disappearance. Whilst for the slowest speed condition, the constant error (mean, ‘accuracy’) was low for all blur levels, for targets with faster speeds, the constant interception error was increased with increasing blur, which resulted in a higher degree of undershooting. The variable interception error (intraindividual standard deviation, ‘precision’) increased as well with increasing blur. Zhao and Warren (2017) conclude that impairing vision by means of blur deteriorates participants’ precision and accuracy (at least for faster speeds) in locomotor interception. Their results are in line with predictions of models including on-line control or continuous updating based on currently available visual information.

Together, these studies certainly show that optical defocus can deteriorate performance in interception tasks, at least for certain levels of blur. Importantly, in all these studies, the dependent measure in interception is actually an amalgam of spatial precision (being in the right place) and temporal precision (at the right time). That is, hits indicate both high spatial and temporal exactitude. Yet, whether misses (i.e., trials in which no successful bat–ball-contact was achieved) were caused by spatiotemporal imprecision or spatial imprecision or temporal imprecision alone was not disentangled.

In fact, according to Recanzone (2009), our visual system is more attuned to spatial perception whereas temporal perception is more precise in the auditory modality. Early evidence for this claim stems from work by O’Connor and Hermelin (1972) who showed that three visually presented digits were mostly analyzed for their spatial localization whilst the same but auditorily presented stimuli were merely regarded concerning their temporal succession. If true, reductions of vision by means of blur should affect spatial perception more severely than temporal perception. Consequently, it is expected that it becomes more difficult to spatially intercept a moving target resulting in a higher spatial variability of the interception response, whilst the temporal response should be less affected. If true, this leads to a more differentiated hypothesis, namely, that a reduction of vision by means of blur should result in a lower spatial precision, but not (or to a lesser extent) in a lower temporal precision. Based on this assumption, the misses observed in the highest blur condition in the study of, for instance, Mann et al. (2007) may have been mainly caused by spatial errors but not so much temporal imprecision. While other variables of interception, like movement time, have been the focus of many studies, only few studies have aimed to disentangle the interception outcome measure in a temporal and spatial (or ‘orthogonal’) response (e.g., Kreyenmeier et al. 2017; Lim 2015). We argue that such a disentanglement would not only be practically relevant but also theoretically insightful when investigating the effect of blur.

To test whether the effect of blur on interception, indeed resulted from diminished spatial and not (or lesser so) temporal precision, in Experiments 1 and 2, participants were asked to indicate (i.e., finger tap) on a large-size touchscreen where and when a virtual ball (moving along parabolic trajectories) crossed a ground line. While in Experiment 1, vision was manipulated using five levels of Gaussian blur, in Experiment 2, we systematically manipulated five levels of contrast instead, to clarify whether coincident changes might have driven the results found for blur.

## Experiment 1

**Fig. 1:**
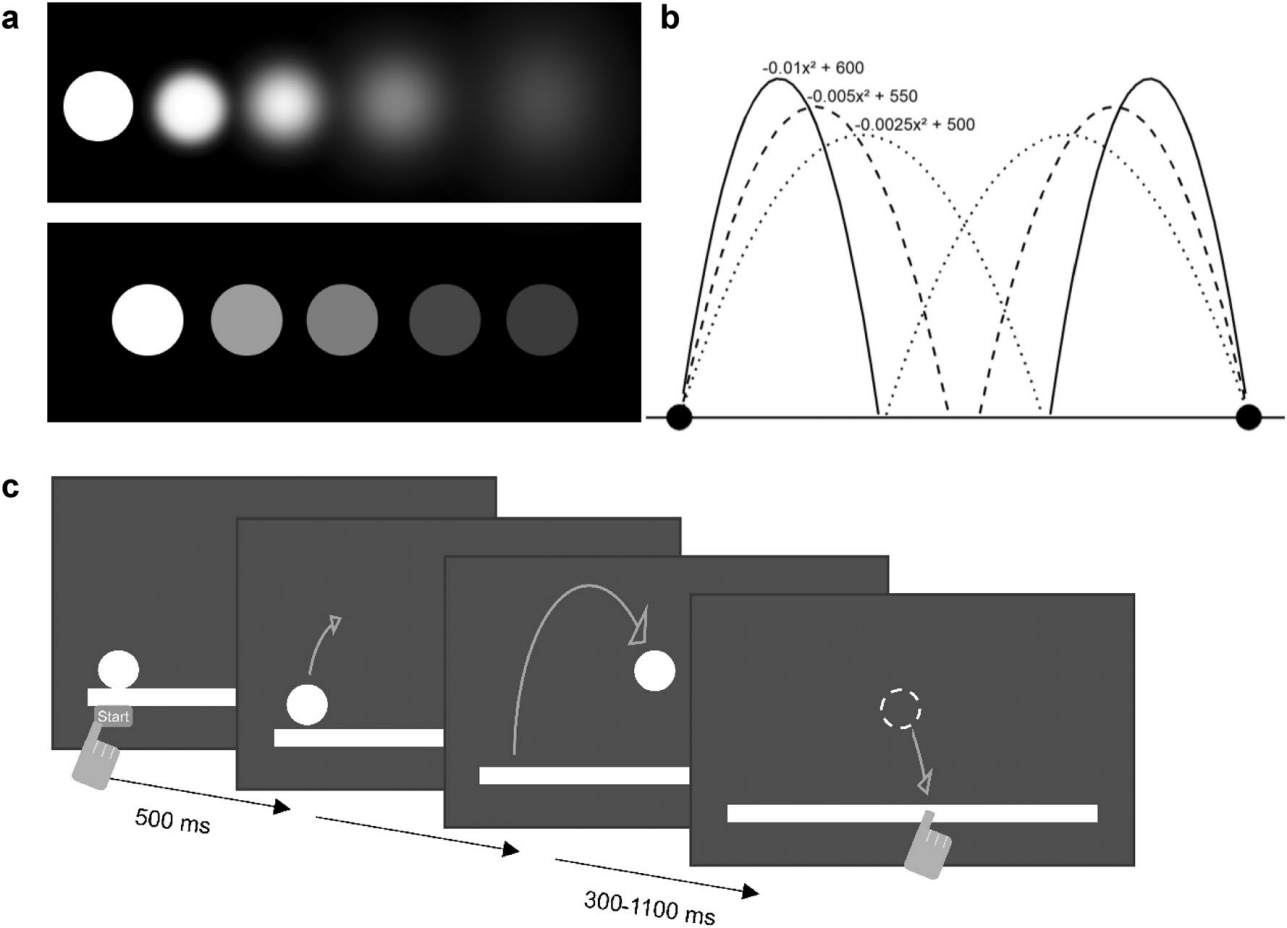
Visual Manipulation and Experimental procedure. **a**: Upper line: Five levels of Gaussian blur (0 px, 10 px, 20 px, 40 px, 60 px) used in Experiment 1. Lower line: Five levels of contrast (95%, 85%, 78%, 46%, 34% Michelson contrast) used in Experiment 2. **b**: Parabola trajectories. **c**: Procedure: After pressing the start button, the ball was presented stationary for 500 ms and then began moving in a parabolic flight curve. 300–1100 ms before it would hit the ground line, it was occluded, and participants had to indicate the location and time of the hit by tapping the location at the right time. The ball’s horizontal velocity was kept constant per trial but altered between trials (3, 4 or 5 px/frame = 8.82, 11.76 or 14.7 cm/s)

To test whether the previously reported effects of (high) blur on interception performance might be caused by reduced spatial and not or lesser so temporal precision in interception, we used a manual interception task on a touchscreen. A virtual ball (white filled circle) was presented moving across the screen in a parabolic flight curve from one side towards the other until it was occluded at different times shortly before hitting a white ground line (for an illustration, see Fig. 1). Participants were asked to intercept the ball by touching the location on the ground line when and where they expected the ball to cross it. Participants’ performance was measured using the spatial constant and variable errors and the temporal constant and variable errors (Tresilian and Plooy 2006). Similar to Brenner et al. (2014) and Zhao and Warren (2017), we interpreted the variable errors as indicators of the respective precision or uncertainty of the response, and the constant errors as accuracy or a general bias in the response (e.g., to overshoot or undershoot the width of the trajectory). Previous research has shown that visual perception is more attenuated towards spatial than temporal information (O’Connor and Hermelin 1972; Recanzone 2009). Reducing vision by blurring the stimulus might therefore have stronger effects on spatial than temporal processing. Consequently, we hypothesized that increasing levels of Gaussian blur of the ball would lead to less precise spatial representations of the stimulus which should result in monotonically decreased spatial precision (as in Zhao and Warren 2017), but would have no effect or a smaller effect on temporal precision. Additionally, the effects of blur on the spatial and temporal constant errors were examined. Query ID="Q3" Text="Please confirm the section headings are correctly identified."

### Materials and methods

#### Participants

A total of 42 participants (15 male, *M*_Age_ = 25.5 years, SD_Age_ = 5.2 years, 40 right-handed) took part in Experiment 1. Seven additionally recruited participants were excluded: three did not fulfil the required visual abilities and four had to be excluded due to technical problems during experimentation [for sample size justification and an a priori power analysis, see Online Resource (OR1)].

Participants were only included in the analysis if they had normal or corrected to normal vision and if they did not report any neurological disorders. To assess vision, two subtests (Acuity C and Contrast C) of the Freiburg Vision Test (FrACT) (Bach 1996, 2006) were conducted (and in the settings the gamma value was set prior to contrast testing). Participants had to reach a visual acuity of 0.00 log MAR or better and a contrast sensitivity of at least 1.7 log CS (Roper and Hassan 2014). Participants received an expense allowance of 8 €. This study forms part of a research program that was approved by the local ethics committee.

#### Materials

We used a 43’’ touchscreen (Iiyama PROLITE TF4338MSC-B1AG, 1920 × 1080, 60 Hz, 2.1 megapixel Full HD, 8 bit, Multi-Touch-Monitor) to present visual stimuli and measure participants’ responses in a manual interception task. The visual stimuli were presented using PsychoPy 3 (Peirce et al. 2019), programmed in the Coder view with a self-written Python script.

In each trial, a white circle (4.9 cm = 100 px diameter) representing a virtual ball was shown on a black screen (see Fig. 1a). The ball moved across the screen following one of three parabola trajectories (see Fig. 1b) mimicking the kinetics of parabolic throwing, however, neglecting air resistance. Hence, the horizontal velocity was kept constant within each trial (3, 4 or 5 px per frame = 8.82, 11.76 or 14.7 cm/s), whilst vertical velocity was varying accordingly. The three trajectories together with the three velocities resulted in nine different transit durations (ranging from 1.63 to 4.97 s; for additional information see OR1 Table 1). Each trajectory started at a white ground line (0.98 × 94 cm) at a distance of 34.3 cm from the center (either on the left or the right) and moved towards the central part of the screen (see Fig. 1b). During the final part, the ball was occluded for 300, 700 or 1100 ms before hitting the ground (Benguigui et al. 2003). The ball was presented in five different blur levels which were manipulated separately using Photoshop’s (“Adobe Photoshop CS,” 2004) Gaussian blur tool with radii of 0, 10, 20, 40 and 60 px (see Fig. 1a upper line). The different levels of all variables were chosen based on Benguigui et al. (2003) and on pilot testing with 11 participants none of whom took part in the main experiment. Different occlusion times, velocities, sides, and trajectories were induced to create different landing positions and times (i.e., induce variability in the task), but were not the focus of analyses.

#### Procedure

The experiment consisted of three parts. After providing informed consent, first participants’ visual acuity and contrast sensitivity were tested using the FrACT (Bach 1996, 2006). Then, in the second and main part, each individually tested participant was asked to sit at approximately 40–50 cm in front of the vertically mounted touchscreen and to perform the manual interception task (see Fig. 1c). The participant began with a block of 12 familiarization trials (without occlusion). Each trial was initiated by the participant pressing the ‘Start’ button presented at the start position of the ball. Upon pressing the button, the ball was presented immediately and started its movement after a 500 ms delay. It moved in a curved trajectory (see Fig. 1b) from the side where the ‘Start’ button was placed toward the central part of the screen.

As illustrated in Fig. 1c, the participants’ task was to indicate with the index finger of their dominant hand when and where they thought the center of the virtual ball (white circle) crossed the ground line (see also Brenner et al. 2013). A touch event was registered as the moment of releasing the finger from the screen. Also, this is what participants should be experienced with due to common touchscreen usage, for instance, on smartphones.^1^ Subsequently, the participant performed two blocks of 12 practice trials similar to the familiarization trials but with occlusion of the ball during the final part of the trajectory. Consequently, the participant had to extrapolate the movement to correctly hit the location and time of crossing. During the familiarization and practice phase trajectories, velocities and occlusion times slightly differed from those used during the main trials of the experiment. After each trial in the familiarization and practice phase, participants received specific feedback about their temporal and spatial error (in ms and mm). Following the familiarization and practice phase, and some additional instructions, the main part of the experiment started.

The ball’s trajectory (3), horizontal velocity (3), occlusion time (3), side (2), and blur levels (5) were altered randomly across the 270 trials (for levels of each variable, see Materials). Every 45 trials, a pause of at least 1 min was included. For motivational reasons, during this pause, accumulated feedback about the previous trials was presented as a percentage score of spatially and temporally correct trials (hit). A hit was defined as touching the screen at a maximum distance of 100 px (4.9 cm) from the current position of the ball’s center. That means that both being spatially and temporally on target was required to count as a hit. In contrast, being at the correct landing position when the ball is currently at its zenith or tapping the correct position when the ball had already passed the ground line was not counted as a hit. Different distances were tested during piloting and a distance of 4.9 cm was chosen to ensure good enough results to keep the motivation of the participants reasonably high (average hit rate between 30 and 40%).

Finally, the participant received a questionnaire collecting information about, for instance, their handedness, age, familiarity with touchscreens, electronic games, and ball sports. The whole procedure lasted about one hour.

#### Data preparation

To analyze the spatial error, only the horizontal deviation (on the ground line) was considered. Based on Zhao and Warren (2017), we took into account the flight direction of the ball (left to right and right to left) when calculating the difference between the location where the participant touched the screen and the actual landing position of the ball. This resulted in coding negative values of the spatial deviation as ‘undershooting’ and positive values as ‘overshooting’^2^ the width of the trajectory.

The temporal deviation was calculated by subtracting the actual time of the ball crossing the ground line from the time when the participant touched the screen (release of the touch event). Hence, positive values signify that the participant touched the screen too late whilst negative values stand for reactions being too early.

Outlier analysis on the level of each individual (Grubbs 1969) indicated that for both dependent measures over 90% of the participants produced at least one outlier. Therefore, outliers defined as all values more than 1.5 times interquartile range above the 75% quantile or below the 25% quantile (on an individual level) were excluded. This analysis resulted in 591 of 11,340 trials (5.2%) for the spatial and 313 of 11,340 trials (2.7%) of the temporal error excluded in Experiment 1, respectively.

The dependent variables were then determined as constant (mean) and variable (standard deviation) errors by aggregating the temporal and the spatial deviation score per participant and blur level (see also Brenner et al. 2014; Tresilian et al. 2009; Tresilian and Plooy 2006; Zhao and Warren 2017). That means that the spatial constant error (spatial accuracy) is defined as the mean difference between the actual location where the ball crossed the ground line and the location where the participant touched the screen, and the spatial variable error (spatial precision) is defined as the within-participant variability (standard deviation) in the spatial interception deviation. Similarly, regarding the temporal response, the mean of each participant (temporal constant error = temporal accuracy) and the within-subject variability (temporal variable error = temporal precision) were computed.

#### Data analysis

To test whether each of the errors (i.e., spatial constant and variable errors; temporal constant and variable errors) differed between blur conditions four separate multilevel models (instead of rmANOVAs, see Field et al. 2013) with error scores per blur level nested in participants were calculated. These models included random intercepts and blur levels as fixed slopes, but no random slopes. To investigate an overall effect of the factor blur, a likelihood ratio test between each model and a corresponding baseline model not including the fixed slopes for blur was calculated (see Field et al. 2013). The code for this test can be found in the OR1 (code 1–3). Significant results were followed-up by post hoc tests (i.e., Tukey Contrasts, see OR1 code 4–6). For significant results, we expected the error score to be monotonically increasing/decreasing with increasing blur levels (similar to the results of Zhao and Warren 2017). To test this, as a second follow-up, additional likelihood ratio tests modeling a linear effect of blur vs. no effect of blur were conducted by defining blur as a numeric variable (instead of a factor).

For the interested reader (and despite not being the aim of our study), the effects of occlusion time, horizontal velocity and side and their interactions with blur on the four dependent variables, as well as associations between the error scores were examined by separate multilevel models and are reported in the Online Resource 1 (OR1, see Fig. 1–8).

For data analysis, R version 3.6.2 (R Core Team 2019) and RStudio version 1.1.456 (RStudio Team 2016) together with the following packages were used: plyr (Wickham 2011), reshape (Wickham 2007), ggplot2 (Wickham 2009), nlme (Pinheiro et al. 2020), dplyr (Wickham et al. 2018), ez (Lawrence 2016), psychReport (Mackenzie 2020), lmerTest (Kuznetsova et al. 2017). A significance level of *α* = 0.05 was used for all analyses.

### Results

On average, participants hit the target in 36.9% of the trials (range 1.5–59%). Overall, participants slightly undershot the target with a spatial constant error of − 9.3 px (− 4.6 mm) and reacted too late with a delay of 0.077 s. The mean spatial variable error was 37.5 px (18.4 mm) and the mean temporal variable error was 0.207 s.

#### Spatial accuracy and spatial precision

**Fig. 2:**
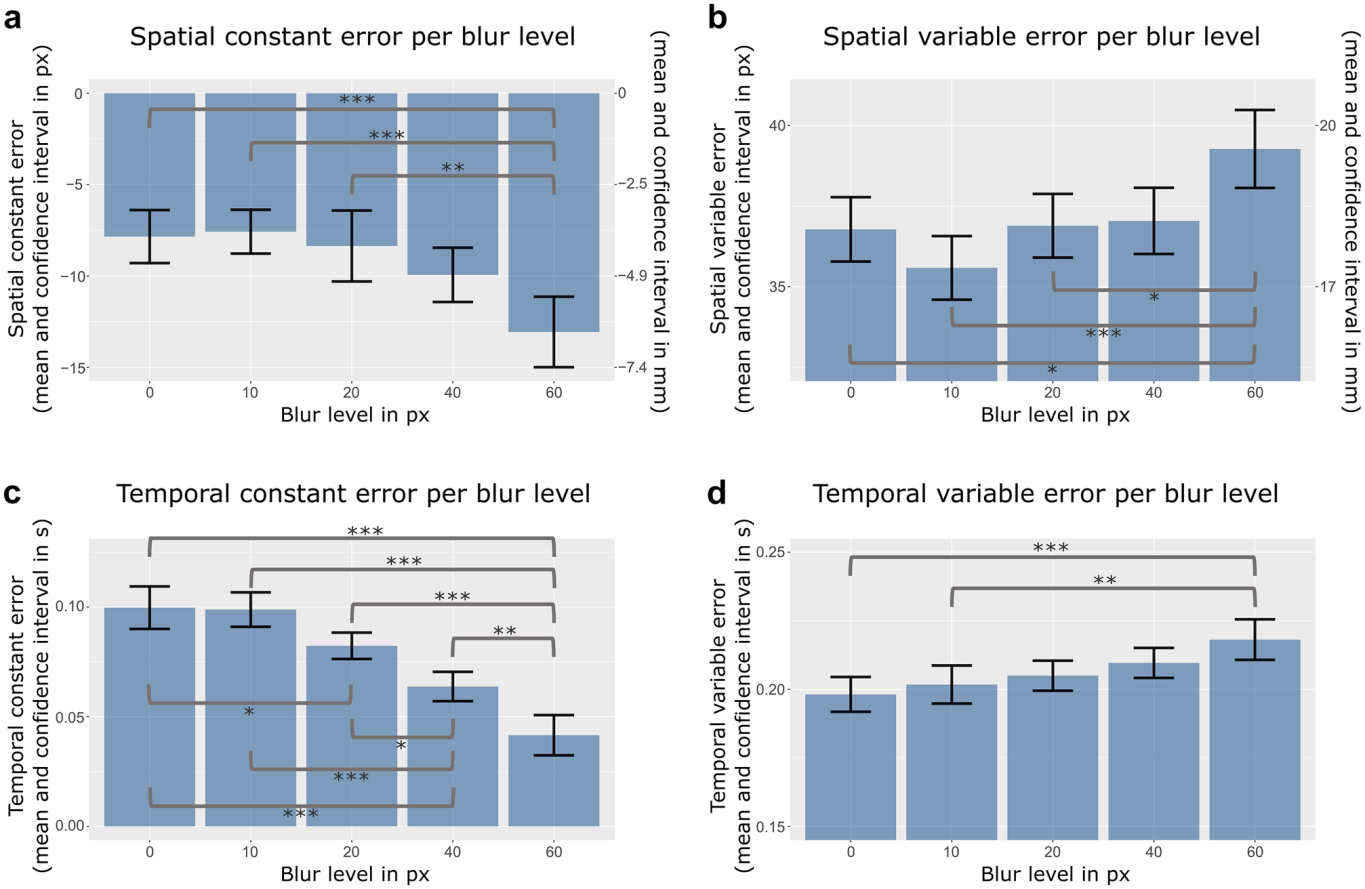
Results of the multilevel analysis: The Effect of visual blur on the spatial variable error (**a**) Spatial constant error (**b**), temporal variable error (**c**), and temporal constant error (**d**). Error bars indicate within-subject confidence intervals adjusted for the within-subject design as suggested by Loftus and Masson (1994)

To test whether blur had an impact on the general bias to overshoot or undershoot the target, the effect of blur on the spatial constant error was evaluated. According to the model comparison, the spatial constant error was significantly affected by different blur levels [*χ*^2^(4) = 29.70, *p* < 0.001]. For post hoc multiple comparisons, see Table 1 (see also Fig. 2a). An additional analysis revealed a significant linear effect of blur on the spatial constant error [*χ*^2^(1) = 26.54, *p* < 0.001], suggesting that participants undershot the target more with increasing blur level and that this relationship did not differ significantly from a linear relationship.

**Table 1:**
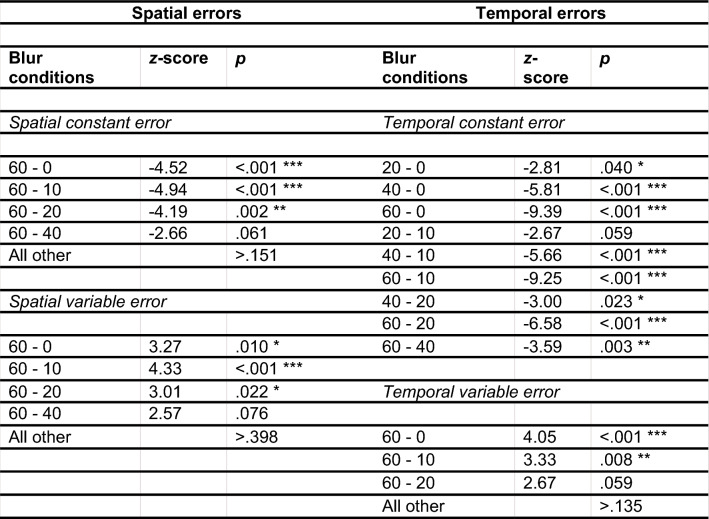
Post hoc analysis for the effect of blur on the four error scores: multiple comparisons of means (Tukey Contrasts)

Only trends and significant differences are reported

Next, it was tested whether the spatial variable error increased with increasing levels of blur. The model comparison revealed a significant effect of blur level [*χ*^2^(1) = 19.55, *p* < 0.001]. As predicted, the more the ball was blurred the bigger the spatial error became (see Fig. 2b and Table 1 for post hoc analyses). The effect for the linear model comparison was significant [χ^2^(1) = 14.93, *p* < 0.001], indicating that the results did not differ significantly from a linear positive relationship between blur and the spatial variable error.

#### Temporal accuracy and temporal precision

It was tested whether blur influenced participants in their general tendency to touch the screen too early or too late. The multilevel model comparison revealed a significant effect of blur on the temporal constant error [*χ*^2^(4) = 95.08, *p* < 0.001]. With increasing blur levels, the mean temporal deviation decreased (= participants reacted earlier, see Fig. 2c). Post hoc analyses revealed significant differences between several blur levels (see Table 1). Again, the linearity of the effect was evaluated with an additional likelihood test. The effect was significant [*χ*^2^(1) = 92.64, *p* < 0.001], further indicating a positive linear relationship between blur and the temporal constant error.

Finally, the temporal variable error was analyzed to examine whether it is affected by blur. There was a significant difference of temporal variable errors between the five blur levels [*χ*^2^(4) = 18.67, *p* < 0.001]. With increasing blur, the temporal variable error increased (see Fig. 2d and Table 1 for post hoc analyses). Additional multilevel analysis with blur as a continuous instead of a factorial variable revealed a significant linear effect of blur on the temporal variable error [*χ*^2^(1) = 18.45, *p* < 0.001].

#### Comparison between temporal and spatial variable error

**Fig. 3:**
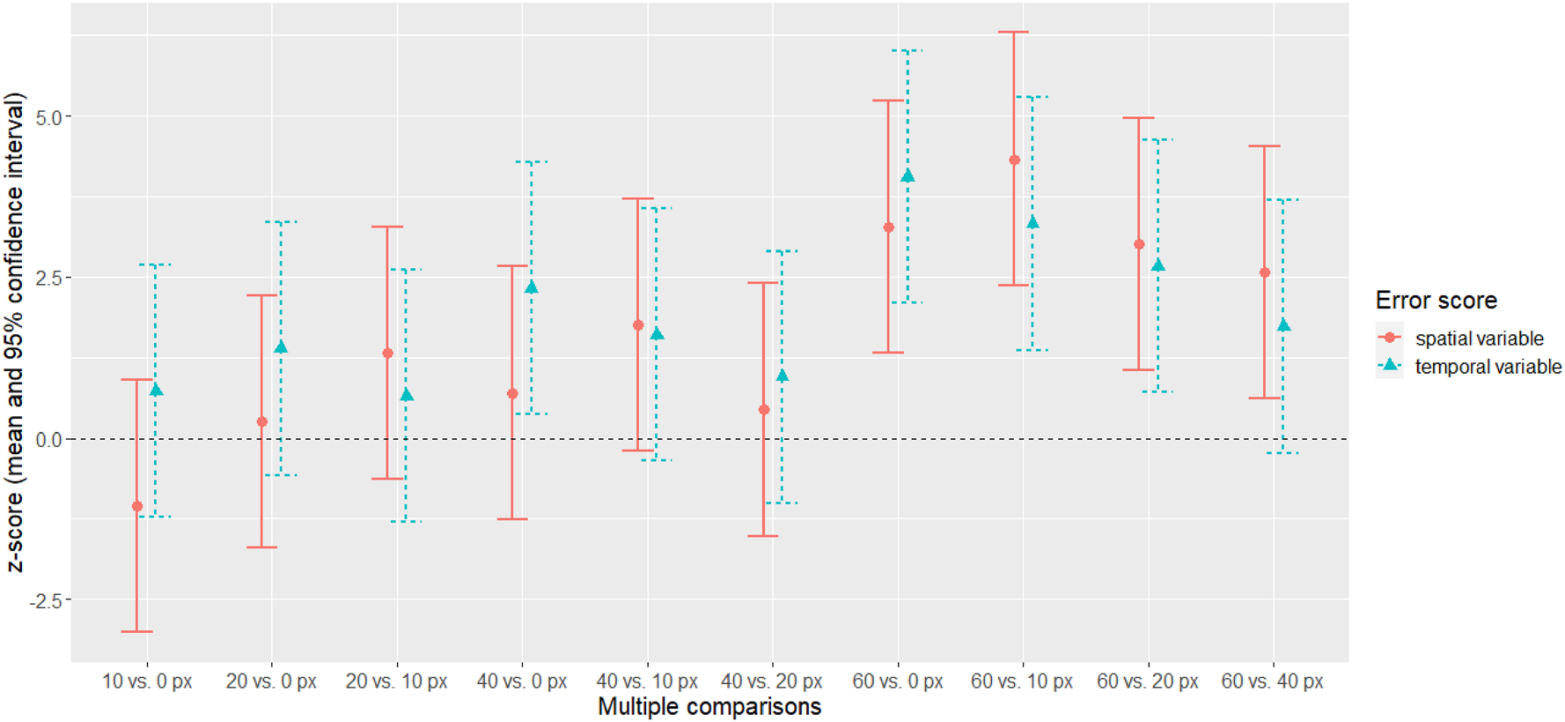
Comparison between effects of blur levels on temporal and spatial variable error. For all multiple comparisons, *z*-scores were compared between the temporal and spatial precision (mean and 95% confidence interval). On the x-axis the respective comparison is specified (e.g.,’10 vs. 0 px’ represents the difference between blur level 10 px vs. blur level 0 px)

To answer the question whether spatial precision is more severely affected by blur than temporal precision, we exploratorily compared the multiple comparisons effect sizes by visualizing the *z*-score (and 95% confidence interval) for both error types (see Fig. 3). Visual inspection showed that there were no significant differences between the temporal and spatial variable error.

### Discussion

The aim of the current study was to disentangle whether the previously reported effects of blur on interception performance were merely produced by reduced spatial in contrast to temporal precision. In agreement with previous research (Mann et al. 2007; Zhao and Warren 2017), we found that (especially very high levels of) blur significantly affected participants’ interception performance and that the effect was negative for most (three out of four) error scores.

First, our results showed that with increasing levels of blur participants’ spatial responses became more variable (less consistent), confirming the notion that the visual system is sensitive to spatial information (O’Connor and Hermelin 1972; Recanzone 2009) and that hence systematic reductions of visual acuity by blurring the target result in reduced spatial precision (increased variable error). Second, in contrast to the hypothesis that reductions of visual acuity should not (or lesser so) affect temporal precision our results showed an additional systematic effect on the temporal variable error. Regarding the *z*-scores of the multiple comparisons for all blur levels, the decreases in spatial and temporal precision are almost identical in size (see Fig. 3). That means that participants temporal responses became less consistent (more variable) with increasing blur in a similar way as their spatial responses. We discuss this discrepancy in more depth in the general discussion and compare our results with previous literature.

There was a negative effect of blur on the spatial accuracy. The spatial constant error was slightly negative for all blur conditions and this general tendency to undershoot the width of the trajectory was increased with increasing levels of blur. Unexpectedly, the temporal accuracy increased with increasing blur levels. Overall, participants overestimated the time the ball would need until crossing the line, but with increasing blur levels this overestimation diminished. This means that participants reacted earlier the more the ball was blurred. This effect might be mediated by coincident changes in perceived size or contrast and will be discussed more thoroughly in the general discussion.

Based on the fact that the manipulations of blur led to coincident changes in contrast (and might as well have altered perceived size), we cannot rule out that some of the results might be mediated by the concomitant changes of the blur manipulation. While there are indications that changes in size do not necessarily affect interception performance (Brenner et al. 2014; Tresilian et al. 2004, 2009), it remains an open question whether changes in contrast might. In fact, decades of research indicate an important role of contrast in vision and related tasks (e.g., Deeb et al. 2015; Johnson and Casson 1995; Thompson et al. 2006), which is why we ran a second experiment in which we systematically manipulated contrast only.

## Experiment 2

Since in Experiment 1, changes in blur were accompanied by changes in contrast, it is possible that some of the effects may have been caused by contrast rather than by blur. It has been shown that reductions of contrast have not only affected vision on the level of visual acuity (Chen and Muhamad 2018; Johnson and Casson 1995), but also reactions towards visual stimuli, for instance, regarding reaction times in visual search tasks (Deeb et al.^2015^) or driving performance (Wood et al. 2014). Contrast sensitivity testing predicts thresholds for the perception of real-world targets (Owsley and Sloane 1987), driving performance (Wood and Owens 2005), and rifle shooting performance (Allen et al. 2018) better than visual acuity testing. Furthermore, research using moving stimuli has shown that perceived speed can be either increased or decreased by low contrasts depending on the actual velocity (Thompson 1982; Thompson et al. 2006; but see also Weiss et al. 2002).

Applying the same task used in Experiment 1, we tested in Experiment 2 whether the effects of blur were mediated by the concomitant changes in contrast, by presenting stimuli of the 0-blur condition but varying contrast levels.

### Materials and methods

#### Participants

A total of 42 participants (12 males, 1 not stated, *M*_Age_ = 21.8 years, SD_Age_ = 2.6 years, 38 right-handed, 1 not stated) took part in the experiment. None of them participated in Experiment 1. Inclusion criteria, expense allowance and ethical approval were the same as in Experiment 1. The sample size was chosen based on the aforementioned a priori power analysis (see OR1).

#### Materials

The materials, procedure and data analysis were the same as in Experiment 1 with only one exception: instead of five levels of Gaussian blur, the ball was presented in five different contrast levels which were matched to the stimuli of Experiment 1. Therefore, the luminance values of the stimuli and the background of Experiment 1 for each blur level were measured with a luminance meter from Gossen (MAVO-SPOT 2) and the Michelson contrast was calculated: 0 px blur = 95%, 10 px blur = 95%, 20 px blur = 93%, 40 px blur = 78%, 60 px blur = 46%. As the contrasts for 0 px, 10 px and 20 px blur were very similar they were summarized as one contrast condition and two more conditions (34% and 85%) were included to keep the design (especially the duration) of the experiment comparable. To summarize, the following Michelson contrasts were used: 95%, 85%, 78%, 46%, 34%, with the ball always being brighter (275 cd/m^2^, 95 cd/m^2^, 62 cd/m^2^, 20 cd/m^2^, 15 cd/m^2^) than the background (~ 8 cd/m^2^). The contrast stimuli for Experiment 2 were generated using GIMP (The GIMP Development Team 2019) (see Fig. 1a bottom line).

After outlier detection, 745 of 11,340 trials (6.6%) for the spatial difference score and 336 of 11,340 trials (3%) for the temporal difference score were excluded in Experiment 2, respectively.

### Results

On average, participants hit the target in 36.2% of the trials (range 11–55%). Across all conditions, participants slightly undershot the landing position of the ball as evidenced by a mean spatial constant error of − 9.2 px (− 4.5 mm). The mean temporal constant error reveals that participants reacted with a delay of 0.094 s on average. The mean spatial variable error was 35.3 px (17.3 mm) and the mean temporal error was 0.180 s.

#### Spatial accuracy and spatial precision

**Fig. 4:**
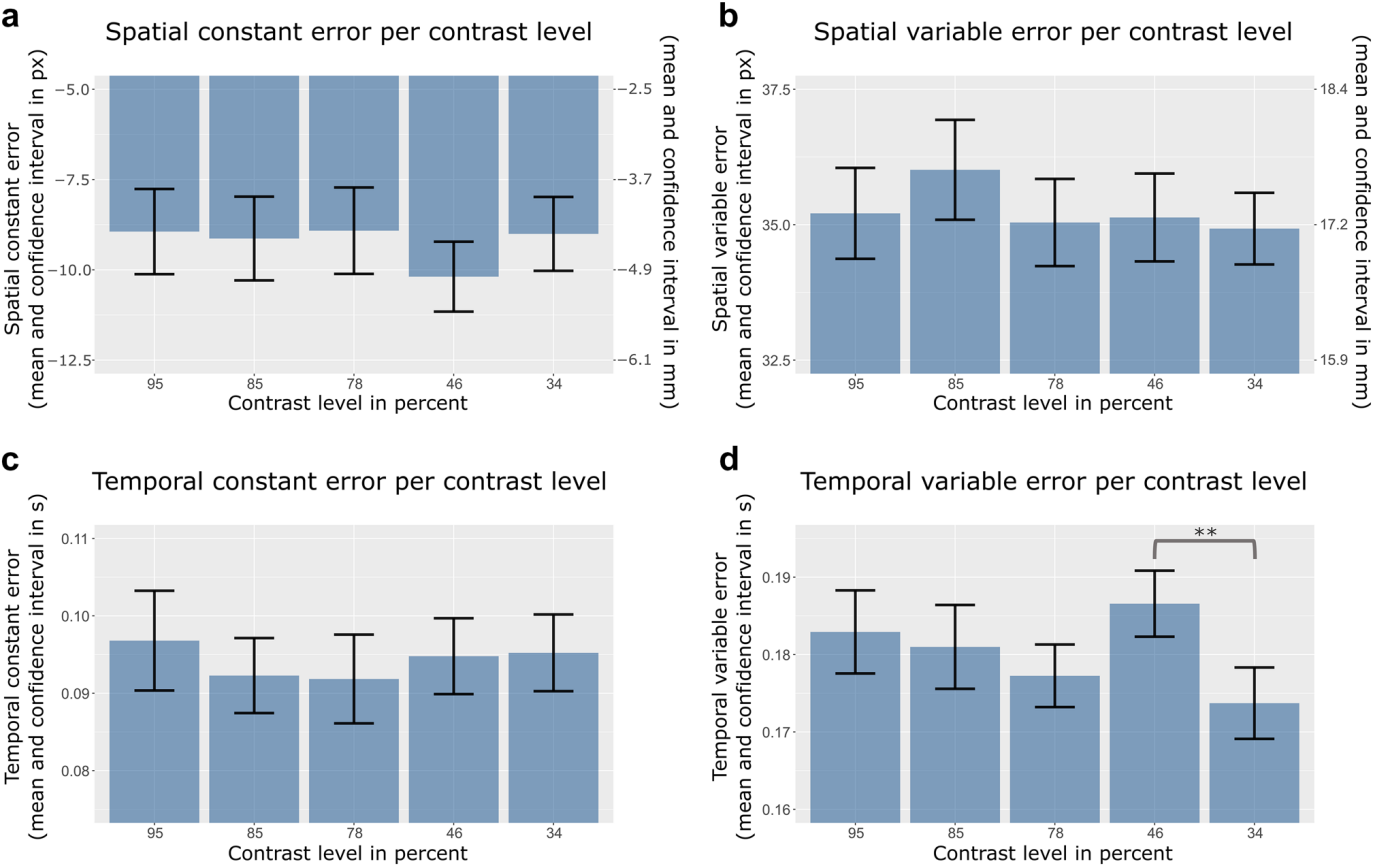
Results of the multilevel analysis: The effect of different contrast levels on the spatial constant error (**a**) Spatial variable error (**b**), temporal constant error (**c**), and temporal variable error (**d**). Error bars indicate within-subject confidence intervals adjusted for the within-subject design as suggested by Loftus and Masson (1994)

The multilevel model comparisons did not reveal any effect of contrast level on the spatial constant error (*p* = 0.534), nor on the spatial variable error (*p* = 0.444). For an illustration, see Fig. 4a, b.

#### Temporal accuracy and temporal precision

According to the multilevel model comparison, there was no effect of contrast level on the temporal constant error (*p* = 0.741). Figure 4c illustrates these results. In contrast, results revealed a significant effect of contrast level on the temporal variable error *χ*^2^(4) = 13.96, *p* = 0.007 (see Fig. 4d). Post hoc analysis (multiple comparisons of Means: Tukey Contrasts) revealed a significant difference between the lowest and the second-lowest contrast level (34 vs. 46%) only (*z* = 3.48, *p* = 0.005). The temporal variable error was higher in the 46% contrast condition. There were non-significant trends for the comparisons of contrasts 95% vs. 34% (*z* = 2.49, *p* = 0.092) and 78% vs. 46% (*z* = − 2.52, *p* = 0.086). All other comparisons did not reach significance (all *p*s > 0.281). There was no evidence of a linear effect (*χ*^2^(1) = 0.93, *p* = 0.334).

### Discussion

To test for contrast as a possible confound or mediator in Experiment 1, in Experiment 2, contrast levels instead of blur were manipulated, and the resulting spatiotemporal interception performance was measured. Changes in contrast did not systematically affect spatial or temporal performance in the applied interception task. There was only one significant but unsystematic effect of contrast on temporal precision indicating less precision for the second-lowest contrast (46% Michelson contrast) compared to the lowest contrast level (34% Michelson contrast). Spatial responses and also temporal accuracy were independent of the contrast level of the ball, contradicting the idea that coincident changes in contrast have caused the results found in Experiment 1.

## General discussion

The aim of the current study was to disentangle the previously reported negative effect of blur on interception performance (e.g., Zhao and Warren 2017) into an effect on spatial vs. temporal precision. Two experiments were run to examine the effect of systematic reductions of the acuity and contrast of a visual stimulus on spatial and temporal precision in a manual interception task. Based on earlier findings indicating a higher sensitivity of the visual system towards spatial when compared to temporal information (O’Connor and Hermelin 1972; Recanzone 2009), we predicted a significant effect of diminished vision on spatial precision but none, or a smaller effect, on temporal precision (both measured as variable errors). Our results seem to only provide partial support for this notion.

### Spatial precision

The results of Experiment 1 showed that participants’ spatial precision indeed decreased with increasing blur. These results of the spatial variable error are in line with previous findings indicating a negative effect of visual blur on visual acuity at the perceptual level (e.g. Johnson and Casson 1995) and on performance measures (e.g. Zhao and Warren 2017).

Especially, the highest blur level caused a significantly reduced precision in comparison with most of the other blur levels corroborating the finding that especially high levels of blur can hamper interception performance (Mann et al. 2007). Yet, there was no effect on spatial precision when using different contrast levels (Experiment 2). Given that the contrast levels were matched to the levels of blur, this suggests that the decrease in spatial precision in Experiment 1 was not due to a coincident decrease in the contrast level when blurring the object. The results of Experiment 2 appear to be in contrast with a number of studies showing significant performance deteriorations with decreasing contrast in visual tasks, such as visual search or target discrimination tasks (Deeb et al. 2015; Owsley and Sloane 1987; Wood et al. 2014; Wood and Owens 2005). To the best of our knowledge, however, our study is the first to have examined the effects of contrast manipulations on manual interception performance. However, it should be noted that the chosen contrast levels were way beyond thresholds and might, therefore, not be appropriate to detect performance differences. As outlined above, the contrast levels were matched to the blur levels in Experiment 1 due to the aim to rule out contrast as a confound or rather mediator. Therefore, the smallest contrast used in the current study was 34%, whereas other studies used also lower levels of 24%, 12%, 6% (Johnson and Casson 1995), or 10% (Thompson et al. 2006).

### Temporal precision

Regarding the manipulations’ impact on temporal precision, the prediction that neither blur nor contrast should affect temporal precision as much as spatial precision, was neither supported by the results of Experiment 1 nor Experiment 2. To start with the latter, in Experiment 2, there was an unsystematic effect on temporal precision. Given that there was no effect for spatial precision, it follows that the results of Experiment 2 do clearly not support the initial hypothesis.

Concerning Experiment 1, blur revealed very similar *z*-scores for both, the temporal and the spatial precision measures (see Fig. 3). Taken at face value, these results seem to suggest that blurring vision impairs temporal precision in a similar way as spatial precision when intercepting a moving target. However, this interpretation would be in conflict with both the theoretical predictions (O’Connor and Hermelin 1972; Recanzone 2009) and previous findings by Brenner et al. (2014) who revealed no effect of blur on temporal precision. There are several possible explanations for this discrepancy: first, the previously reported results may not generalize or transfer to our interception task. In contrast to most of the studies investigating the effect of blur on performance, the current task was conducted on a touchscreen. This might impose different demands on the subject compared to, for instance, intercepting a real ball with a cricket or baseball bat (e.g., Brenner et al. 2014; Mann 2010). Second, the effects may depend on the way blur was induced. In contrast to others, we used image processing (Gaussian blur) to blur only the target instead of lenses (e.g., Brenner et al. 2014; Bulson et al. 2008, 2015) or contact lenses (e.g., Mann et al. 2007; Mann 2010) blurring whole vision. When using lenses, the distance between the target and the observer plays an important role: clarity increases with decreasing distance. In our study, distance was held approximately constant, and the amount of blur was the same throughout a trial. We believe that blurring whole vision might impose completely different demands on the participant: in our design, there was a clearly visible ground line, indicating the ‘landing position’ and thereby defining the time, when the participant had to tap the screen. If that line would have been blurred, too, identifying this landing position might have become more difficult, because the exact point might be represented less precisely. That means, participants would not have known when to tap because of a spatial problem: localizing the ground line. In other words, this might have resulted in a temporal error which may not have been caused by an error in motion prediction or interceptive action, but rather by the less clear spatial location of the ground line. Third, the effects might be mediated by a third factor, namely, potential concomitant changes in the target’s perceived size. Blurring means that the boundaries visually fade out resulting in a less clearly defined size. That means that the outer points of the ball were more widely distributed the more it was blurred but as well the background intruded more with increasing blur. If only the outer points were taken as a criterion, this might have led to the perception of increased size (but note that this was neither tested nor self-reported by any of the participants). If so, it might have been more difficult to identify the center of the ball, which was important to fulfil the temporal part of the task (i.e., to intercept the target when its center crossed the ground line). That means that a predominantly spatial problem (identifying the center) resulted in a temporal effect (reduced temporal precision). Future research should examine and control for such effects by checking whether the center is indeed less precisely identified in blurred objects (e.g., in a stationary task). Fourth, previous studies have shown that visual manipulations can systematically impact velocity perception (Gegenfurtner and Hawken 1996), which would, contrary to our hypothesis, result in temporal errors in the current task. A detailed analyses and discussion of velocity effects can be found in the OR1. In short, in both experiments, participants intercepted more delayed for faster velocities, but no effects on the temporal variable errors were evident. If the chosen blur manipulations indeed change the perception of velocity this might be reflected in changes in temporal accuracy.

### Spatial accuracy

In both experiments, we found a general tendency to horizontally undershoot the spatial location of the target at the interception point. In Experiment 1, this tendency increased with increasing levels of blur, whereas in Experiment 2, contrast had no effect on the spatial accuracy. The effect of blur is in congruence with the findings of Zhao and Warren (2017) who reported that blurred stimuli led to more undershooting than less blurred stimuli.^3^ However, the overall undershooting conflicts with predictions from extrapolation research (Fulvio et al. 2015), showing that when occluding curved trajectories participants either predict locally linear or locally quadratic continuations, none of which would lead to undershooting in the current task.

### Temporal accuracy

Consistently in both experiments, participants showed delayed reactions towards the moving stimulus. This general tendency might be explained by the incapability of humans to use acceleration information for their time to arrival estimation (Benguigui et al. 2003) and interception performance (Brenner et al. 2016). During the occluded part of the trajectory vertical velocity increases, but participants should be unable to predict this increase, at least if they are not able to learn from previous trials with the same acceleration (Brenner et al. 2016). This should lead to delayed reactions as found in both experiments of the current study and consistent with the findings of Brenner et al. (2016) who showed delayed reactions when the time point of tapping was clearly defined as in the current paradigm and not free to choose. Additional results supporting this notion can be found in the OR1 when discussing the effects of occlusion times on the temporal accuracy. Interestingly, in Experiment 1, blur significantly affected the size of the delay, whilst contrast in Experiment 2 had no effect.

Based on the argumentation Zhao and Warren (2017), that increasing levels of blur imply reduced spatial frequencies and that, therefore, the object should appear to move slower than a less blurred one (Brooks et al. 2011; Diener et al. 1976; Smith and Edgar 1990), one would expect that perceived reduced speed (increasing blur) should lead to more delayed responses. Yet, the opposite was the case: with increasing blur, the temporal accuracy (constant error) was decreased meaning that participants’ overestimation of the ball’s movement time diminished. We argue that this finding is not necessarily questioning the assumption of perceived reduced speed but might instead be resolved by one of following potential explanations: first, it is conceivable but still unlikely that blurring may—perhaps somewhat counterintuitively—have facilitated participants’ interception performance. Second, despite thorough instructions participants might have not reacted towards the center of the ball but instead (unintendedly) attended the ‘edge’ of the ball. The more the ball was blurred the closer to the ground its outer points appeared (before occlusion) and the earlier they would have crossed the line (during extrapolation). If participants attended the ‘edge’ of the ball, they might have pressed earlier with increasing blur, because the outer points of the ball were spread wider. Third, it is possible that participants associated specific blur levels with specific ball types that implied characteristics like mass. A recent study has shown that time to contact estimations depend on the mass of a visual stimulus probably due to explicit heuristics or even implicit conclusions from mass to falling speed (Vicovaro et al. 2019).

In Experiment 2, no effects of reduced contrast where found, indicating that the results of blur were not due to changes in contrast which is in line with a study on time to contact estimations that found no effect of contrast, or luminance levels (Landwehr et al. 2013). Yet, a vast amount of studies showing altered velocity perception for moving stimuli with low contrast levels would predict effects on temporal accuracy (Feldstein and Peli 2020; Thompson 1982; Thompson et al. 2006). For instance, Battaglini et al. (2013) found a main effect of contrast levels on speed perception. They showed that decreasing contrast leads to an underestimation of target speed (even during occlusion) which should result in delayed interception responses in the current paradigm. As explained above, this discrepancy might be due to the relatively high contrast levels used in the current experiment (and potentially also the experiments in Landwehr et al. 2013).

### Additional factors and limitations

The current study was specifically designed to analyze the effect of manipulations of blur on interception performance in a task simulating a ball flight curve. Obviously, there is a vast number of other factors found to impact performance in interception, for instance, concerning properties of the task (cf., Bosco et al. 2012; Brenner and Smeets 2009; Brouwer et al. 2000, 2005; Tresilian et al. 2003; Tresilian and Houseman 2005) or participants’ characteristics (e.g., fatigue, Barte et al. 2020; amount of stabilization, Couto et al. 2020; sports experience, Yu and Liu 2020). While the investigation of interindividual differences was not part of the current study, some task-related factors (stimulus velocity, side, and occlusion time) were manipulated to produce variability. Full insight about additional separate analyses and their discussion are provided in the OR1. Note in this regard that these factors were not of central interest to our research question. Since no 0 s occlusion condition was included in the experiment, our study design does not allow and hence cannot dissociate whether the effects found for blur result from a misperception of the visible part of the trajectory or erroneous extrapolation during the occluded part. Nonetheless, it should be noted that studies on time to contact and speed estimations reported common underlying mechanisms (Battaglini et al. 2018) and electrophysiological correlates for visible and occluded targets (Makin et al. 2009).

As explained above, in the current design, perceived target size might be a factor mediating the effect of blur. Previous interception research reveals no consensus about effects of target size: for instance: in a batting task measuring interception performance as temporal error, Brenner et al. (2014) found no effect of different ball sizes. In interception tasks using a manipulandum, Tresilian et al. (2004) and Tresilian et al. (2009) found no consistent main effect of target size on movement time, the spatial variable error, or the constant error, but on maximum movement speed. In contrast, Brouwer et al. (2005) and Tresilian and Houseman (2005) revealed a significant effect of target size on movement time. These results indicate that certain aspects of interceptive actions (like movement time) can be influenced by the size of the target, but often, specifically the spatial and temporal errors were not affected. To conclude, we cannot rule out that increases in perceived target size (if they were present) might have affected the reported results. However, the above-mentioned literature does not provide clear evidence for this hypothesis. Future research should focus on the impact of such task-related factors and the possible moderators of and interactions with blur. Furthermore, it might be advantageous to investigate interindividual differences in the temporal and spatial performance measures, as studies indicate that, for instance, sports experience (e.g., Yu and Liu 2020) and the amount of stabilization (learning, e.g., Couto et al. 2020) might impact participants’ performance.

If not due to substantial differences in the task demands or the way of blurring, the effects of blur on both temporal measures in Experiment 1 might be due to space-time-associations in interception tasks that we aimed to disentangle. Despite our experimental rigor to disambiguate spatial and temporal contributions to the motor response, temporal estimates of the ball’s movement were not completely independent of spatial perception. That is, to predict when the ball’s center would cross the ground line, participants needed to perceive its location at certain timepoints. Therefore, when spatial precision was diminished due to noisier spatial representations during presentation and/or extrapolation, temporal precision should be affected as well. One result supporting this notion is the positive association between temporal and spatial difference scores found in both experiments (see OR1). This is in contrast to the often-found trade-off between temporal and spatial responses in interception paradigms (e.g., Tresilian et al. 2009). The current results seem to suggest that temporal and spatial responses were not perfectly independent of each other in the applied paradigm.

It should be mentioned that in contrast to other studies (e.g., Brenner and Smeets 1997) participants’ heads were not fixated using a chin rest. We did not use a chin rest to allow participants to rotate their head and ensure optimal conditions for interception performance (Mann et al. 2019). Though participants were asked to keep their head at a distance of approximately 50 cm from the screen, it is possible that participants have slightly moved their head (back and/or forth). Therefore, we could not specify and report visual angles with certainty, and hence refrained from doing so. In future studies the use of chin rests might be advisable, if one aims for better experimental control at the costs of less ecologically valid interception performance or if the movement range of the stimuli is relatively small. Furthermore, modelling air resistance and gravitation forth of the earth within the target’s motion might help to improve the ecological validity of future studies (as in Kreyenmeier et al. 2017; Vicovaro et al. 2019).

In reaching and grasping tasks, an important theory has emerged from research on the contributions of the ventral and dorsal visual pathways, referred to as the two visual systems or dual-pathway theory (Goodale et al. 1991; Goodale and Milner 1992; Milner and Goodale 1995). According to this theory, the predictions regarding the effects of blur and contrast on an interception task would have been very different. More specifically, the dual-pathway theory claims that there are two visual streams within visual processing. One, the ventral pathway (‘what’), functions to create a conscious percept of the visual stimulus, while the other, the dorsal pathway (‘where’), is thought to work in a more goal-directed fashion, sub-consciously guiding our actions. Based on the differences regarding their innervating properties, concrete hypotheses about effects of blur or contrast on different types of tasks have been postulated. In short, the dorsal pathway only includes magnocellular input which is characterized by fast processing and high sensitivity towards contrast, whereas the ventral pathway is characterized as a magno- and parvocellular system including slower transmission with high spatial resolution and colour-sensitivity. These physiological differences suggest that blur, as a reduction of the spatial resolution, should first and foremost impact processing within the ventral, but not necessarily the dorsal pathway (Norman 2002). On the other hand, decreases in contrast should have an effect mainly on action-based tasks, requiring more dorsal information processing. In general, an interception task as applied in the current study is thought to be a goal-directed, mainly dorsally processed task. Consequently, performance in interception tasks should be mainly affected by changes in contrast and not by changes in blur. Our findings are seemingly not in line with these predictions. Interestingly, however, recent research calls into question the clear distinctions between the two pathways (Milner and Goodale 2008) and more recent research has shown that the systems tend to interact (e.g., Cañal-Bruland et al. 2013). If true, this interaction may explain our findings, that blur affected a supposedly highly action-directed and therefore dorsally processed interception task, whilst contrast did not (see also Mann 2010), at least regarding the chosen levels. Indeed, as participants had some time to observe the object before it was occluded and reached the ground line, there might have been enough time for the slower parvocellular system to process all information and for both streams to interact. Moreover, to investigate the role of visual input for interception performance it might be advisable to include eye tracking in future studies, as recent interception studies suggest close associations between eye and hand movements and confirm the important role of eye movements on interception responses (de la Malla et al. 2017; Fooken et al. 2016, 2021; Kreyenmeier et al. 2017).

On a final note, we deem it likely that both response modality and the modality of stimulus presentation play important roles in determining spatial and temporal precision in manual interception (see also Loeffler et al. 2018). As concerns response modality, future research may be well advised to compare different ways to respond, for example, by contrasting verbal vs. motor responses. Regarding the modality of stimulus presentation, future research about the differences in sensitivity towards spatial and temporal information may also focus on the auditory modality, as it has been shown that the auditory system is more dominated by temporal than by spatial information (O’Connor and Hermelin 1972; Recanzone 2009). It follows that another way to test the hypothesis we sought to shed light on in this paper, may be to manipulate the quality of auditory information, thereby testing the counterpart of the hypothesis, namely that reductions of auditory qualities should more strongly affect temporal than spatial precision (Tolentino-Castro et al. 2021).

In summary, in two experiments, we tested whether participants’ spatial precision would suffer more severely from visual manipulations of blur (Experiment 1) and contrast (Experiment 2) than temporal precision in a manual interception task. Whilst contrast had no systematic effect on neither error score, blurring the moving object reduced both spatial and temporal precision similarly.

## Supplementary Information

Below is the link to the electronic supplementary material.

Supplementary file1 (DOCX 3834 KB)

## Data Availability

Please contact the first author (annaschroeger@gmail.com).

## Footnotes

1 It allows for spatial adjustments before the finger is released from the screen and the timing is recorded. Pilot testing showed that participants only shortly touched the screen and did not make any obvious spatial adjustments during the contact. Participants were informed about and had time to familiarize with the task demands during the initial 24 familiarization and practice trials (with immediate spatial and temporal feedback). Importantly, participants almost never used the possibility to spatially adjust and instead only shortly touched the screen.

2 Instead of a general left/right coding. Please note that analyses for each side separately revealed similar effects.

3 Please note that their measure (‘spatial accuracy’) was still an amalgam of spatial and temporal accuracy. Therefore, an undershooting effect might as well be treated as an ‘too early’ reaction.

## Code availability

Please contact the first author (annaschroeger@gmail.com).
