# Supplementary material for: Effects of visual blur and contrast on spatial and temporal precision in manual interception
Source: Exp Brain Res. 2021 Sep 4;239(11):3343–58. doi: 10.1007/s00221-021-06184-8 (PMC8542000; doi:10.1007/s00221-021-06184-8)
Supplement: Supplementary file 1 — Supplementary file1 (DOCX 3834 KB) [file 221_2021_6184_MOESM1_ESM.docx]

Online Resource 1 (OR1)

Article title:

**Effects of visual blur and contrast on spatial and temporal precision in manual interception**

Journal name: Experimental Brain Research

Author names:

Anna Schroeger^a^*

J. Walter Tolentino-Castro^b^

Markus Raab^b,c^

Rouwen Cañal-Bruland^a^*

^a^Department for the Psychology of Human Movement and Sport, Institute of Sport Science, Friedrich Schiller University Jena, Germany

^b^Department of Performance Psychology, Institute of Psychology, German Sport University Cologne, Germany

^c^School of Applied Sciences, London South Bank University, UK

*Correspondence concerning this article should be addressed to Anna Schroeger (annaschroeger@gmail.com).

Materials and Methods

Power analysis

The sample size of 42 was chosen based on an a priori power analysis (5 repeated measures MANOVA, within factors) using G*Power 3.1 (Faul et al., 2007; Faul et al., 2009) with an estimated effect size of *f*= .18 (small effect of η² = .03), an alpha = .05, a high power = 0.8 and a correlation among repeated measures of *r* = .5.

Target motion

The ball’s movement was defined by the parabola equations and the velocity in horizontal direction. The resulting distance and duration can be taken from Table 1 in the Online Resource 1 (OR1).

Table 1. Additional information of the stimuli.

| **Velocity in x-direction *v_x,start_*** | **Parabola equation** | **a in 1/px** | **d in px** | **Distance in x-direction** | **Duration** |
| --- | --- | --- | --- | --- | --- |
| 3 px/frame | -0.01x² + 600 | 0.01 | 245 | 490 px = 24.0 cm | 2.72 s |
| 4 px/frame | -0.01x² + 600 | 0.01 | 245 | 490 px = 24.0 cm | 2.04 s |
| 5 px/frame | -0.01x² + 600 | 0.01 | 245 | 490 px = 24.0 cm | 1.63 s |
| 3 px/frame | -0.005x² + 550 | 0.005 | 332 | 663 px = 32.5 cm | 3.69 s |
| 4 px/frame | -0.005x² + 550 | 0.005 | 332 | 663 px = 32.5 cm | 2.76 s |
| 5 px/frame | -0.005x² + 550 | 0.005 | 332 | 663 px = 32.5 cm | 2.21 s |
| 3 px/frame | -0.0025x² + 500 | 0.0025 | 447 | 894 px = 43.8 cm | 4.97 s |
| 4 px/frame | -0.0025x² + 500 | 0.0025 | 447 | 894 px = 43.8 cm | 3.73 s |
| 5 px/frame | -0.0025x² + 500 | 0.0025 | 447 | 894 px = 43.8 cm | 2.98 s |

The following equations describe the targets motion:

$$v_{x}\left( t \right)=v_{x, start}$$

$$v_{y}\left( t \right)= v_{y, start}-gt$$

$$g=2av_{x}^{2}$$

$$v_{y, start}= dav_{x}$$

Code for main analysis

The following code was used for multilevel model likelihood ratio test of the main analyses reported in the main manuscript:

baseline <- lme(errorscore ~ 1, random = ~1|participant/blur, data = data, method = "ML") (1)

model <- lme(errorscore ~ blur, random = ~1|participant/blur, data = data, method = "ML") (2)

anova(baseline, model) (3)

postHocs<-glht(model, linfct = mcp(level = "Tukey")) (4)

summary(postHocs) (5)

confint(postHocs) (6)

In addition to the main analyses of blur and contrast, the effects of occlusion time, velocity and flight direction were calculated. Separate multilevel models including one of those variables, blur (Experiment 1) or contrast (Experiment 2) and the interaction between both were run. All variables were included as continuous variables and fixed slopes, but random intercepts were modeled (see also Field et al., 2013). Previous research has shown that each of those variables might impact interception performance (e.g., Bosco et al., 2012; Brenner et al., 2014; Tresilian et al., 2009).

To evaluate whether temporal and spatial errors may reflect two independent entities of the intercepting action, we additionally explored the presence of associations between the spatial and temporal deviation variables (measured as absolute values). Using multilevel modeling of the effect of the spatial difference on the temporal difference variable, we compared a random intercept fixed slope model with a random intercept (no slope) model to look for a general relationship between those two variables. To examine potential interindividual differences in this relationship, we additionally compared a random intercept random (and fixed) slope model with a random intercept fixed slope model.

We correlated the participants spatial and temporal errors with each of the variables measured within the questionnaire. Depending on data distribution, Pearson’s correlation coefficient or Kendall’s rank correlation coefficient are reported.

Finally, to analyze performance between experiments we compared all error scores for the no-blur and highest-contrast condition between both experiments.

Experiment 1 – blur

Results

Table 2 in the OR1 provides an overview about the error scores per blur level, occlusion time, horizontal velocity, and side.

Table 2. Descriptive data of task performance. Means (and standard deviations) of each error score per factor level are reported. Negative values of the spatial accuracy indicate that participants undershot the trajectory whilst positive values represent overshooting. Concerning the temporal accuracy, positive values indicate that the participants’ temporal response was too late.

| **Variable** | **Spatial**  **accuracy** | **Spatial**  **precision** | **Temporal accuracy** | **Temporal precision** |
| --- | --- | --- | --- | --- |
| **Blur** |  |  |  |  |
| 0 px | -7.73 px (20.36) | 36.97 px (13.82) | 0.100 s (0.206) | 0.198 s (0.071) |
| 10 px | -7.20 px (21.60) | 36.15 px (13.12) | 0.099 s (0.216) | 0.202 s (0.072) |
| 20 px | -8.14 px (21.49) | 37.18 px (10.51) | 0.082 s (0.208) | 0.205 s (0.071) |
| 40 px | -10.06 px (21.41) | 37.52 px (11.62) | 0.064 s (0.198) | 0.210 s (0.068) |
| 60 px | -13.40 px (22.33) | 39.53 px (13.42) | 0.041 s (0.215) | 0.218 s (0.074) |
| **Occlusion time** |  |  |  |  |
| 0.3 s | -1.33 px (12.11) | 23.57 px (13.15) | 0.103 s (0.184) | 0.153 s (0.067) |
| 0.7 s | -9.60 px (21.28) | 36.05 px (13.17) | 0.103 s (0.216) | 0.199 s (0.078) |
| 1.1 s | -17.54 px (32.49) | 45.61 px (11.80) | 0.027 s (0.251) | 0.225 s (0.070) |
| **Horizontal velocity** | | | |  |
| 3 px/frame | -10.66 px (19.43) | 32.05 px (13.05) | 0.004 s (0.230) | 0.198 s (0.071) |
| 4 px/frame | -8.86 px (21.11) | 38.05 px (11.97) | 0.087 s (0.207) | 0.202 s (0.059) |
| 5 px/frame | -8.23 px (23.81) | 41.48 px (12.13) | 0.142 s (0.198) | 0.198 s (0.060) |
| **Side** |  |  |  |  |
| Left to right | -10.20 px (24.89) | 35.55 px (11.24) | 0.075 s (0.203) | 0.209 s (0.072) |
| Right to left | -8.28 px (21.83) | 36.80 px (13.47) | 0.080 s (0.212) | 0.207 s (0.066) |

Spatial constant error

For the additional analysis only the models including either velocity or occlusion time revealed significant effects. There was a significant linear effect of velocity on the spatial constant error [*χ*²(1) = 4.95, *p* = .026]. With increasing velocity participants undershot the trajectory less. Another finding is that when including velocity as predictor, the significant linear effect of blur disappeared [*p* > .723] and there was no significant interaction between blur and velocity [*p* > .430]. The model including occlusion time revealed a significant main effect of occlusion time [*χ*²(1) = 76.29, *p* < .001], and blur [*χ*²(1) = 8.29, *p* = .004], but no significant interaction between them [*p* > .192]. With increasing occlusion time, participants undershot the trajectory more severely. The model for side showed no main effect of side and no interaction between blur and side [all *p*s > .303], while the main effect of blur remained significant [*χ*²(1) = 11.65, *p*< .001]. For an illustration see Fig. 1 in the OR1.


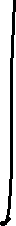

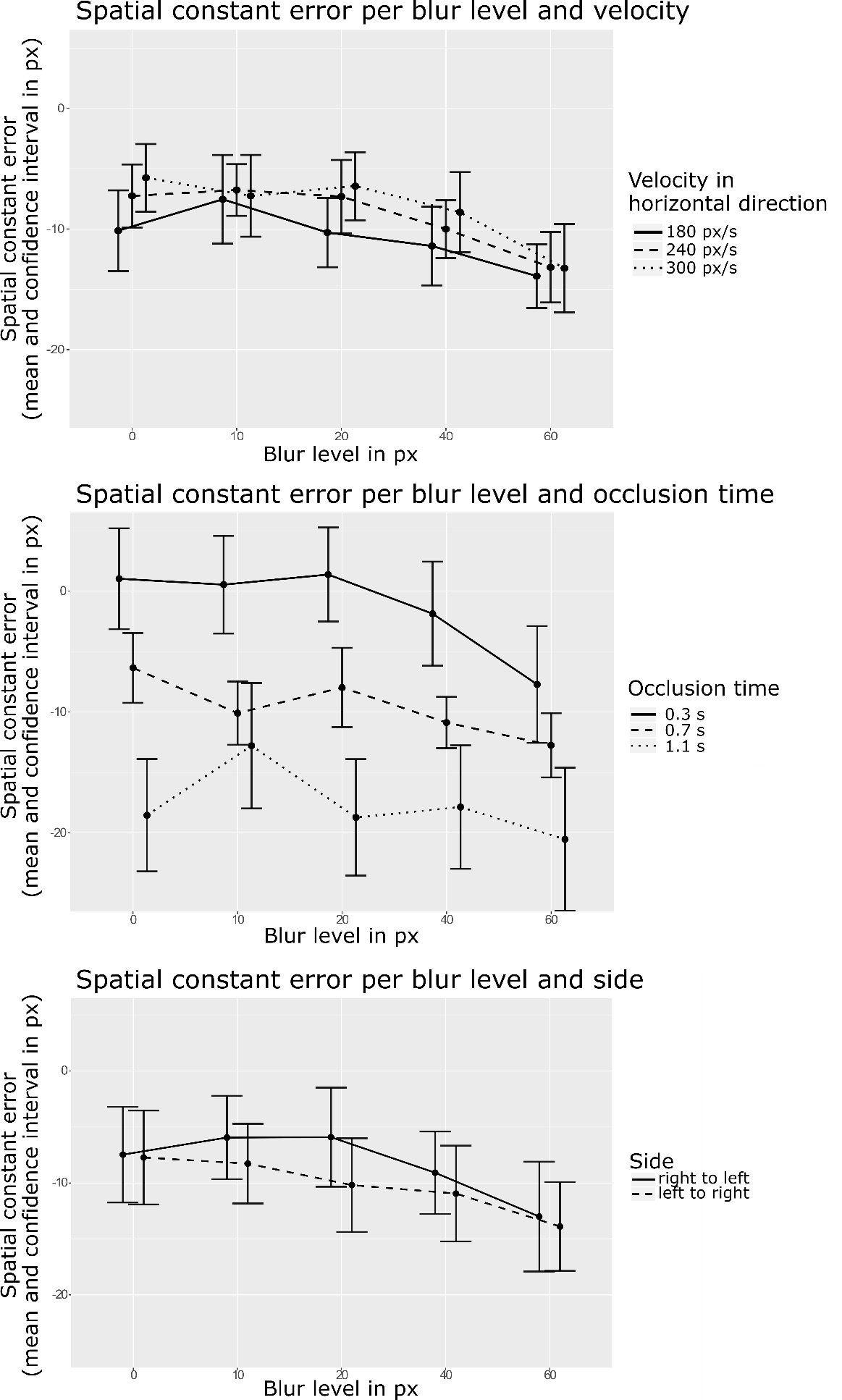


**Fig. 1** Effects of velocity (a), occlusion time (b), and side (c) on the spatial constant error per blur level. Displayed are means and confidence intervals per condition

**a**

**c**

**b**

Spatial variable error

There was a significant effect of velocity [*χ*²(1) = 94.45, *p* < .001], and blur [*χ*²(1)  = 7.74, *p* = .005], and a significant interaction between velocity and blur [*χ*²(1) = 4.40, *p* < .036]. Both variables increased the spatial variable error, but there was also a negative interaction. Both, higher amounts of blur [*χ*²(1) = 17.86, *p* < .001], and occlusion time [*χ*²(1) = 388.96, *p* < .001], increased the spatial variable error. Furthermore, there was a significant interaction between occlusion time and blur [*χ*²(1) = 6.47, *p* = .011]. With increasing occlusion time, the effect of blur decreased. When including side, only the effect of blur reached significance [*χ*²(1) = 11.37, *p* < .001, all other *p*s > .107]. Those results are illustrated in Fig. 2 in the OR1.


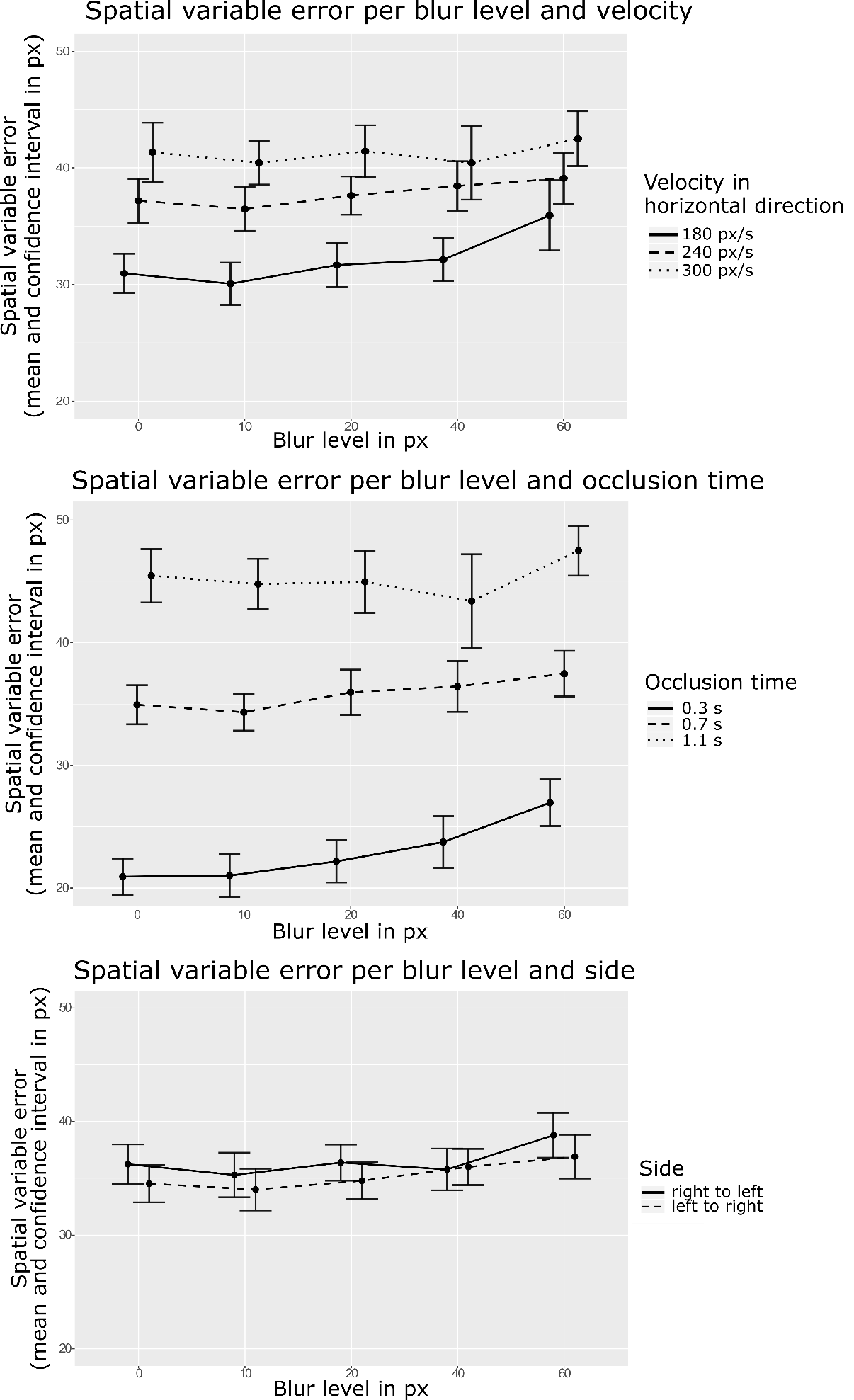


**Fig. 2** Effects of velocity (a), occlusion time (b), and side (c) on the spatial variable error per blur level. Displayed are means and confidence intervals per condition

**a**

**b**

**c**

Temporal constant error

With increasing velocity, the temporal constant error increased significantly [*χ*²(1) = 170.41, *p* < .001]. Furthermore, blur still significantly decreased the temporal constant error [*χ*²(1) = 17.53, *p* < .001], and there was a significant interaction [*χ*²(1) = 5.38, *p* = .020]. With increasing velocity, the effect of blur was decreased. The model for occlusion time revealed a significant main effect of occlusion time [*χ*²(1) = 52.52, *p* < .001], and a significant main effect of blur [*χ*²(1) = 19.72, *p* < .001], on the temporal constant error, but the interaction missed significance [*p* = .064]. Both blur and occlusion time let to earlier responses (resulting in an increased temporal accuracy). Side had no significant main effect nor interaction with blur [all *p*s > .564], but there was a significant main effect of blur [*χ*²(1) = 120.38, *p* < .001]. These results are depicted in Fig. 3 in the OR1.


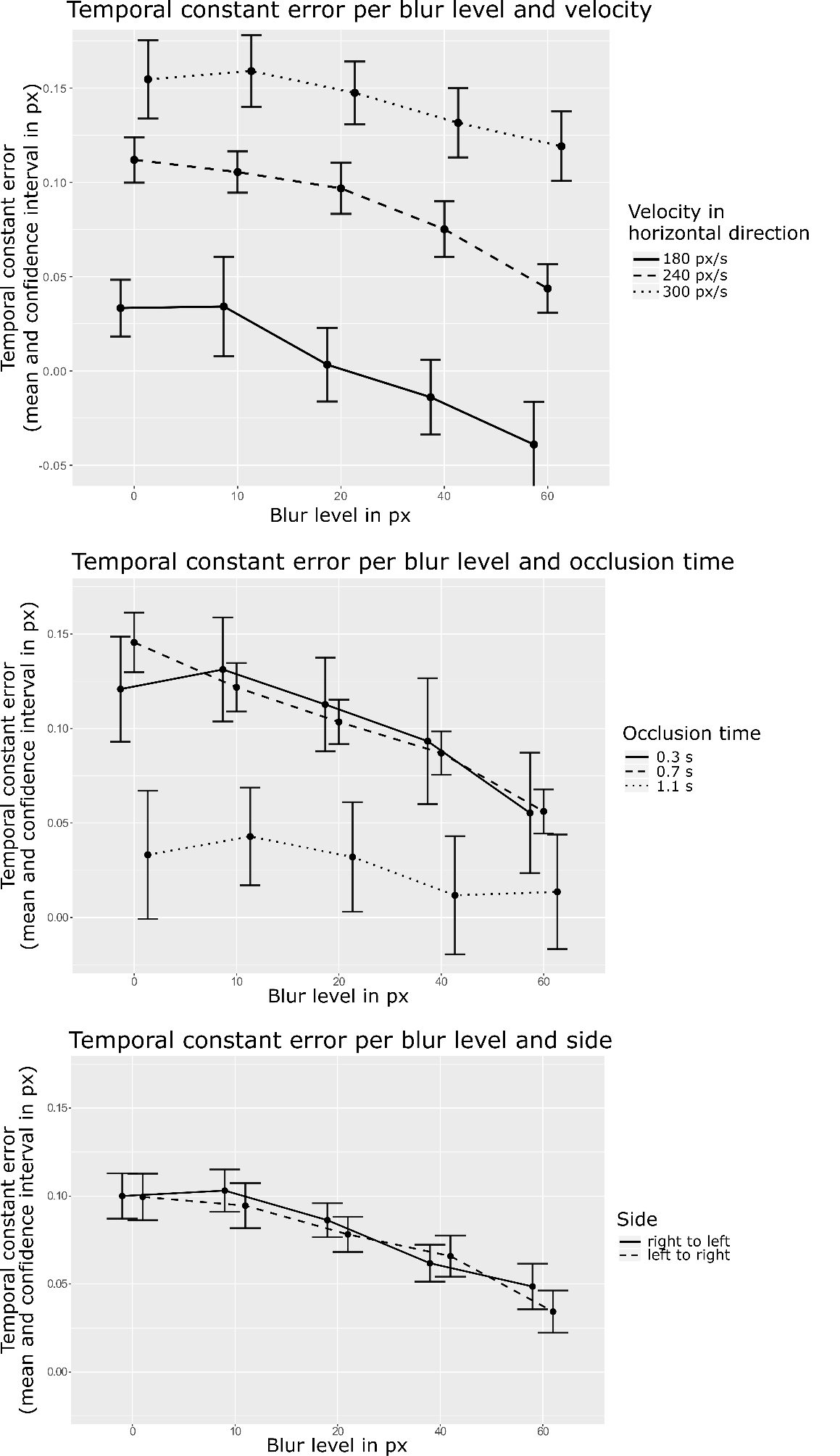


**Fig. 3** Effects of velocity (a), occlusion time (b), and side (c) on the temporal constant error per blur level. Displayed are means and confidence intervals per condition

**a**

**c**

**b**

Temporal variable error

For the model including random intercepts and fixed effects of blur, velocity and their interaction, there was neither a significant main effect of blur nor of velocity [all *p*s > .199], but a significant interaction between both [*χ*²(1) = 4.22, *p* = .040]. When adding occlusion time as predictor instead, the temporal variable error increased with increasing occlusion time [*χ*²(1) = 106.19, *p* < .001], but the analysis did not reveal a significant effect of blur [*p* > .251] nor a significant interaction between blur and occlusion time [*p* > .316]. Please note, that the temporal variable error was neither affected by side nor by the interaction between side and blur [all *p*s > .667], but there was a significant main effect of blur [*χ*²(1) = 23.60, *p* < .001]. Please see Fig. 4 in the OR1 for an illustration.


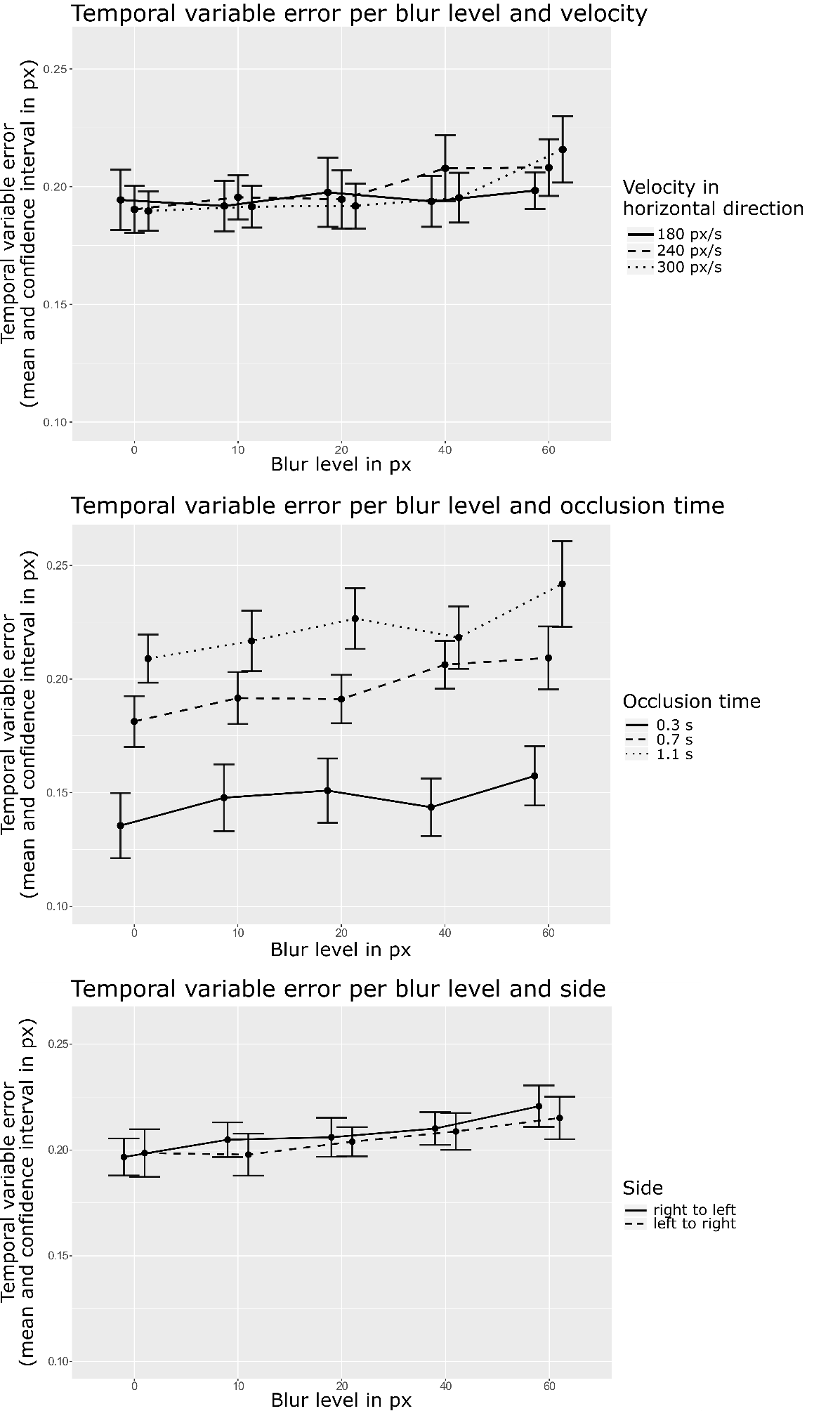


**Fig. 4** Effects of velocity (a), occlusion time (b), and side (c) on the temporal variable error per blur level. Displayed are means and confidence intervals per condition

**a**

**c**

**b**

Association between errors

To evaluate whether the two error types are associated with each other, we ran additional analyses. There was a significant positive fixed effect of the spatial difference score on the temporal difference score [*χ*²(1) = 319.91, *p* < .001]. With increasing spatial deviation participants’ temporal deviation increased (on a trial level). Furthermore, there was a significant random slope indicating interindividual variability in the relationship between the spatial and temporal deviation [*χ*²(2) = 150.14, *p* < .001]. These findings indicate a relationship between the temporal and the spatial deviation, but the strength of this relationship varied between individuals.

Correlation with constant errors

Regarding the data acquired via the questionnaire, there was a small but significant correlation between how many hours participants played electronic games per day and the temporal constant error [*τ* = .24, *z* = 1.99, *p* = .046]. The error increases with an increasing number of playing hours. Additionally, there was a non-significant trend for a correlation between age and the spatial constant error [*τ* = .20, *z* = 1.76, *p* = .078]. With increasing age, the spatial constant error increased. There were no other significant correlations (considering experience with electronic games on a touchscreen in hours per day, age in years, visual performance on acuity and contrast sensitivity, ball sport training in hours per week, touchscreen familiarity in hours per day, all *p*s > .158).

Correlation with variable errors

There were no significant correlations between the variable errors and the data of the questionnaire [all *p*s > .213].

Experiment 2 – contrast

Results

Table 3 in the OR1 provides an overview about the error scores per contrast level, occlusion time, horizontal velocity, and side.

Table 3. Descriptive data of task performance. Means (and standard deviations) of each error score per factor level are reported. Negative values of the spatial accuracy indicate that participants undershot the trajectory whilst positive values represent overshooting. Concerning the temporal accuracy, positive values indicate that the participants’ temporal response was too late.

| **Variable** | **Spatial**  **accuracy** | **Spatial**  **precision** | **Temporal accuracy** | **Temporal precision** |
| --- | --- | --- | --- | --- |
| **Contrast** |  |  |  |  |
| 95% | -8.94 px (25.43) | 35.21 px (7.56) | 0.097 s (0.130) | 0.183 s (0.041) |
| 85% | -9.13 px (24.90) | 36.01 px (8.94) | 0.092 s (0.126) | 0.181 s (0.036) |
| 78% | -8.92 px (26.03) | 35.04 px (8.42) | 0.092 s (0.131) | 0.177 s (0.036) |
| 46% | -10.19 px (24.69) | 35.13 px (7.57) | 0.095 s (0.132) | 0.187 s (0.036) |
| 34% | -9.00 px (25.13) | 34.93 px (7.78) | 0.095 s (0.128) | 0.174 s (0.038) |
| **Occlusion time** |  |  |  |  |
| 0.3 s | 2.33 px (12.88) | 18.98 px (4.04) | 0.115 s (0.097) | 0.123 s (0.026) |
| 0.7 s | -10.10 px (26.57) | 32.33 px (7.08) | 0.125 s (0.131) | 0.171 s (0.033) |
| 1.1 s | -20.94 px (39.15) | 42.11 px (8.74) | 0.041 s (0.182) | 0.202 s (0.045) |
| **Horizontal velocity** | | | | |
| 3 px/frame | -8.35 px (19.99) | 29.89 px (5.27) | 0.046 s (0.129) | 0.175 s (0.036) |
| 4 px/frame | -9.88 px (26.45) | 36.10 px (8.12) | 0.097 s (0.130) | 0.176 s (0.036) |
| 5 px/frame | -9.40 px (29.86) | 38.41 px (8.88) | 0.140 s (0.131) | 0.176 s (0.035) |
| **Side** |  |  |  |  |
| Left to right | -7.53 px (26.51) | 33.60 px (7.84) | 0.094 s (0.125) | 0.178 s (0.035) |
| Right to left | -10.86 px (27.48) | 34.07 px (7.21) | 0.094 s (0.133) | 0.182 s (0.035) |

Spatial constant error

The spatial constant error was not affected by velocity, contrast, or their interaction [all *p*s > .595]. With increasing occlusion time, participants undershot the target more [*χ*²(1) = 28.09, *p* < .001], but contrast and the interaction of contrast and occlusion time had no significant effect [all *p*s > .803]. When adding side as predictor, the effect of contrast and the interaction of contrast and side did not show any significant effect [all *p*s > .371], and the effect of side slightly missed significance [*χ*²(1) = 3.20, *p* = .074], indicating more undershooting when the trajectory started at the right side. For an illustration, see Fig. 5 in the OR1.


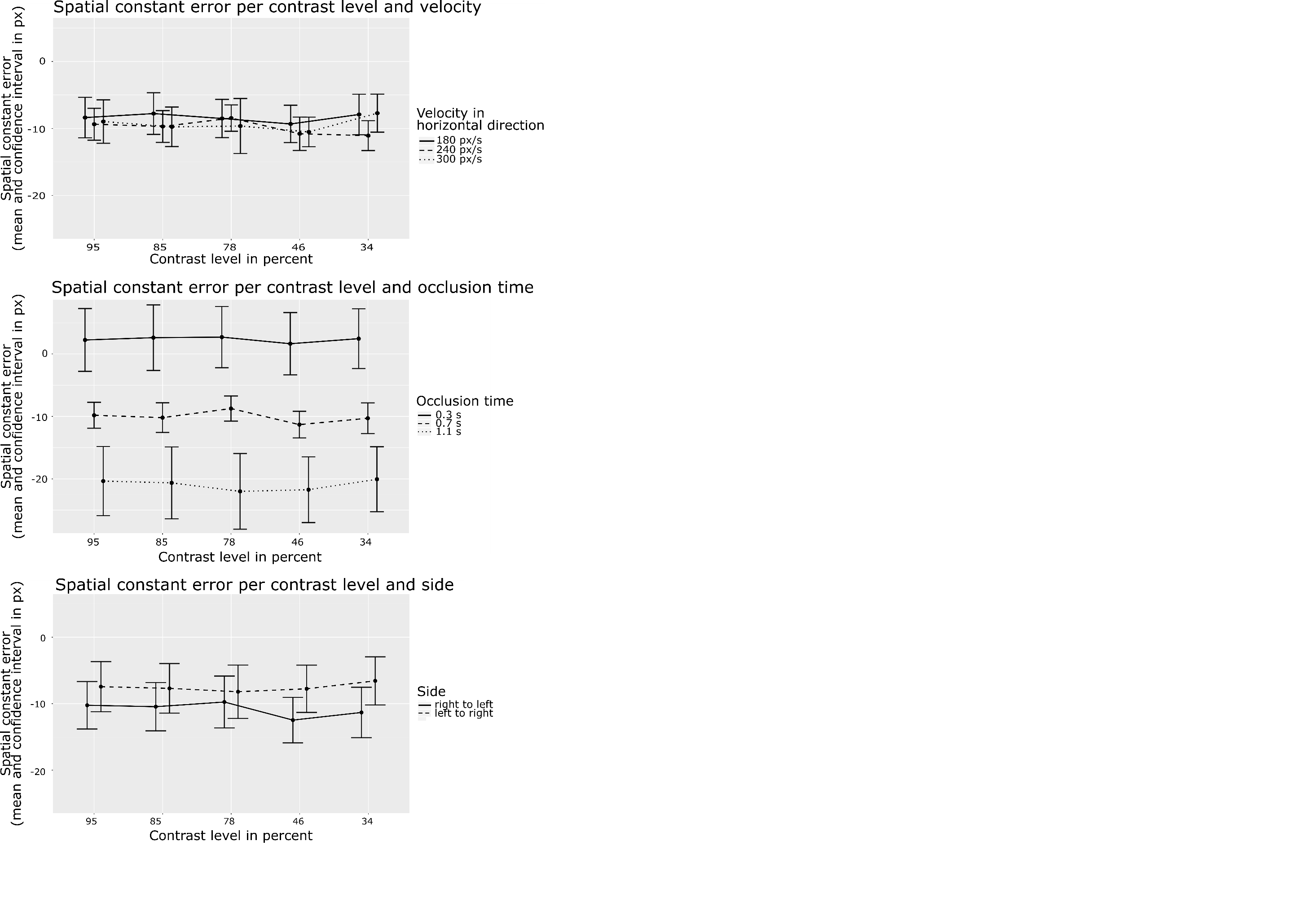


**Fig. 5** Effects of velocity (a), occlusion time (b), and side (c) on the spatial constant error per contrast level. Displayed are means and confidence intervals per condition

**a**

**b**

**c**

Spatial variable error

The spatial variable error was significantly increased with increasing velocity, *χ*²(1) = 42.68, *p* < .001]. There was a non-significant trend that the spatial variable error decreased with decreasing contrast [χ²(1) = 3.46, p = .063], and that this effect decreased with increasing velocity [χ²(1) = 2.89, p < .089]. The model with occlusion time and contrast, indicated a significant main effect for occlusion time only [*χ*²(1) = 118.72, *p* < .001, all other *p*s > .222]. The spatial variable error increased with increasing occlusion time. When side was included as predictor, none of the main effects nor the interaction were significant [all *p*s > .152]. The effects of the additional factors on the spatial variable error are depicted in Fig. 6 in the OR1.


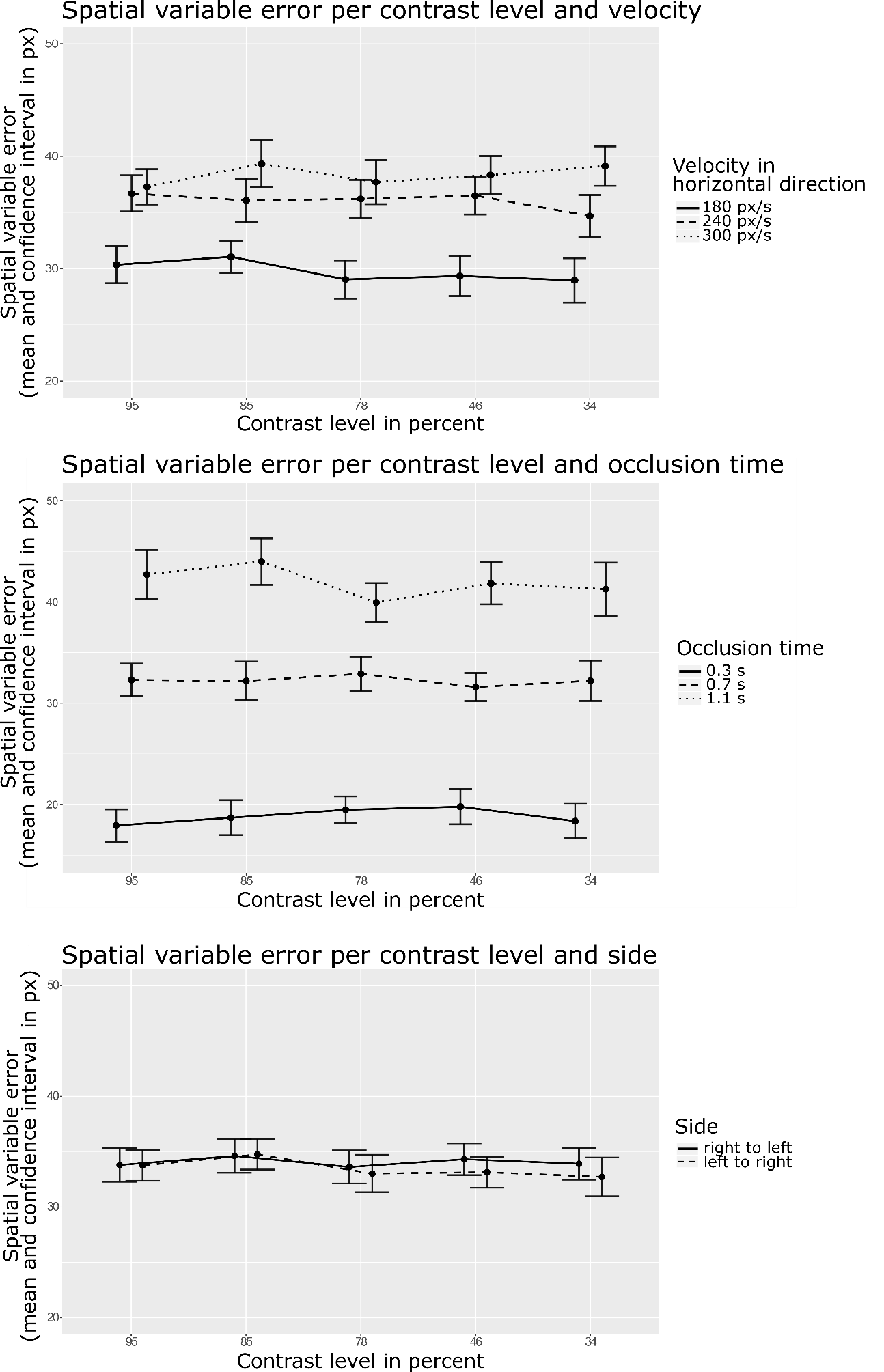


**Fig. 6** Effects of velocity (a), occlusion time (b), and side (c) on the spatial variable error per contrast level. Displayed are means and confidence intervals per condition

**a**

**b**

**c**

Temporal constant error

The model of velocity revealed a significant positive main effect of velocity on the temporal constant error [*χ*²(1) = 67.15, *p* < .001], but no other significant effects [all *p*s > .630]. When adding occlusion time instead, the temporal constant error decreased (earlier reactions) with increasing occluded intervals [*χ*²(1) = 10.73, *p* = .001]. The other two effects were not significant [all *p*s > .884]. A model including side, contrast and the interaction of both did not reveal any significant results [all *p*s > .515]. Please see Fig. 7 in the OR1 for an illustration.


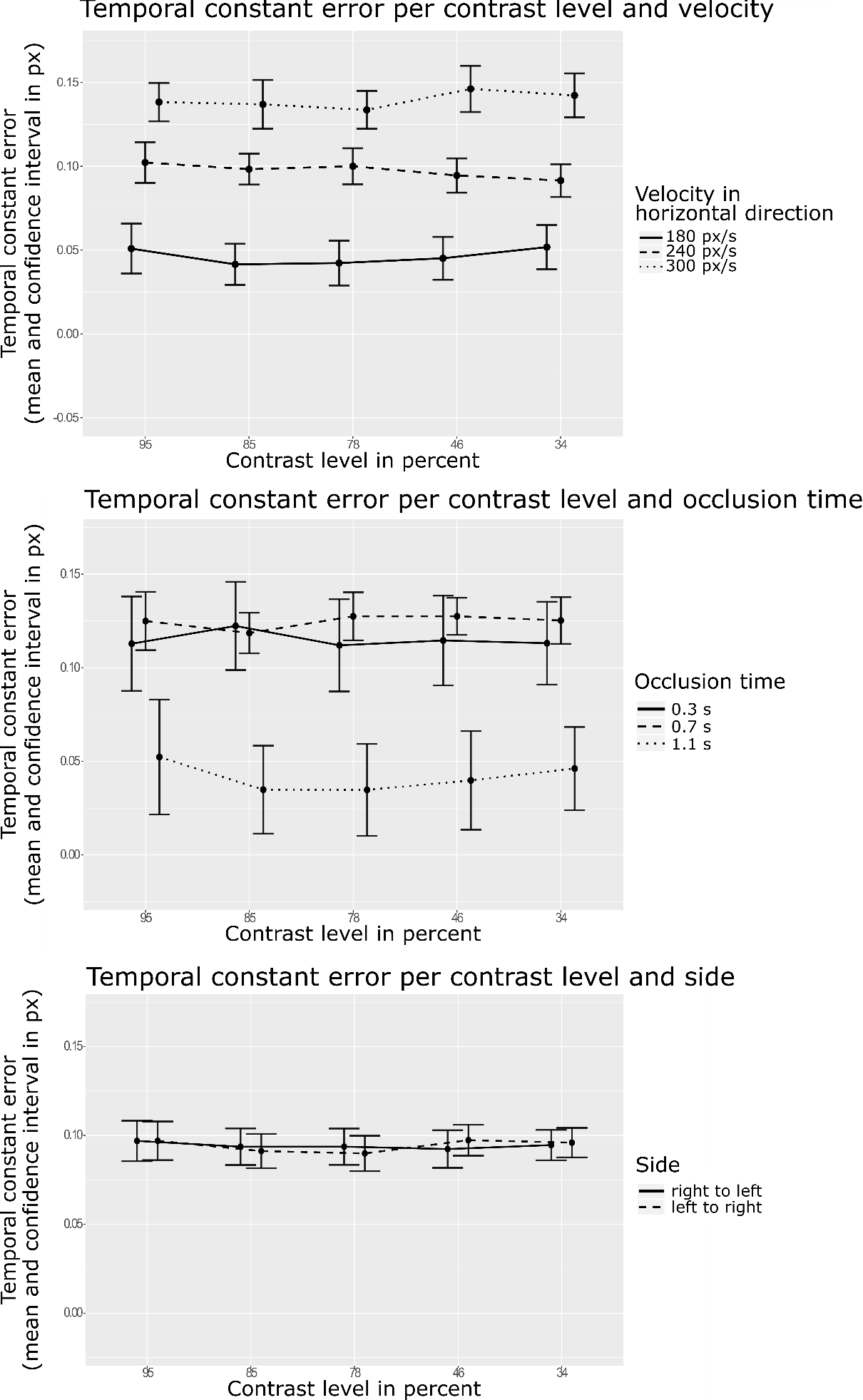


**a**

**b**

**c**

**Fig. 7** Effects of velocity (a), occlusion time (b), and side (c) on the temporal constant error per contrast level. Displayed are means and confidence intervals per condition

Temporal variable error

When including velocity as predictor, none of the effects reached significance [all *p*s > .0714]. The model including occlusion time revealed no effects for contrast and the interaction between contrast and occlusion time [all *p*s > .842], but a significant main effect of occlusion time [*χ*²(1) = 62.17, *p* < .001]. The longer the occluded interval was, the more variable participants’ responses became. There was no significant effect of side, contrast, or the interaction between both [all *p*s > .415]. These results are visualized in Fig. 8 in the OR1.


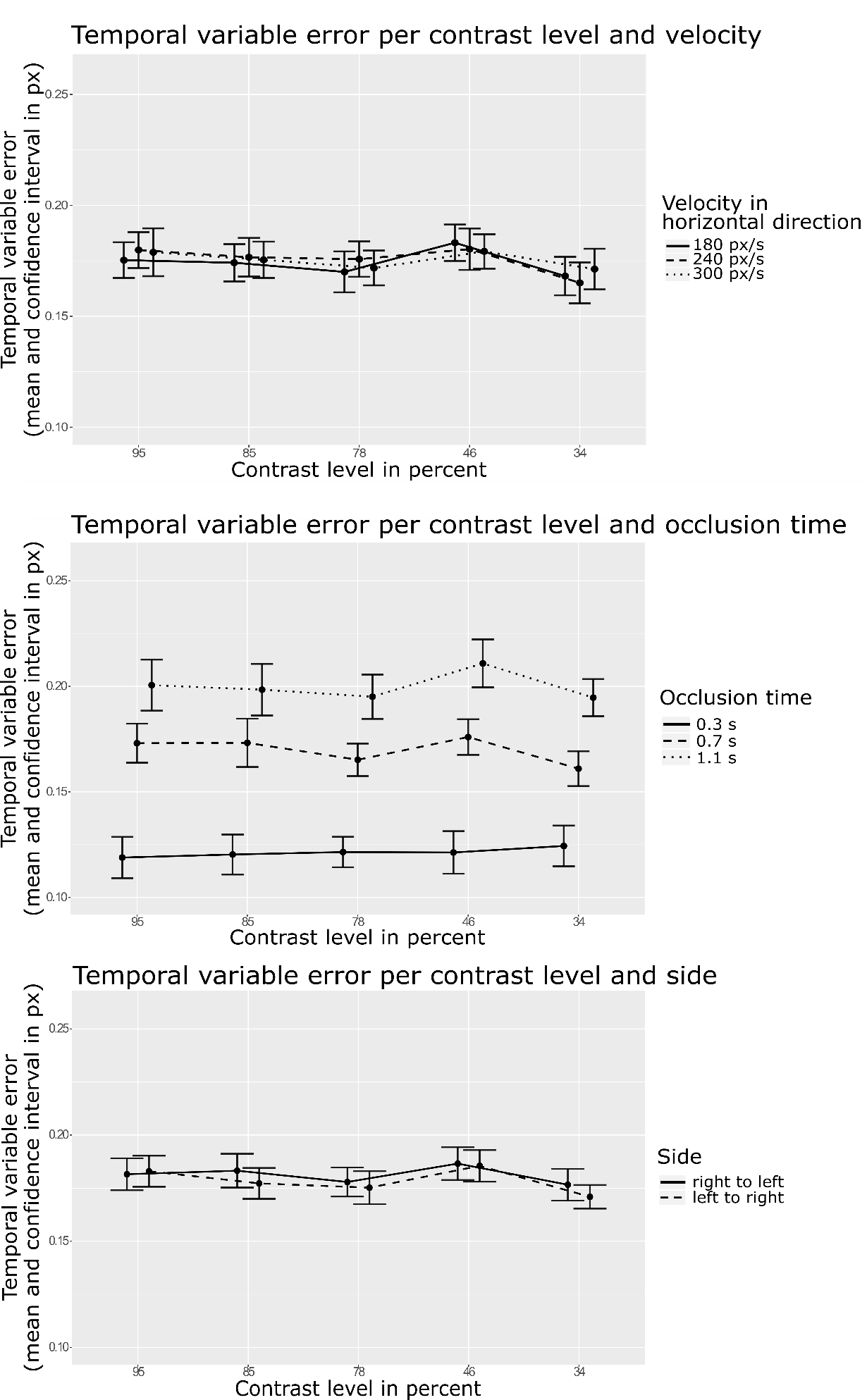


**Fig. 8** Effects of velocity (a), occlusion time (b), and side (c) on the temporal variable error per contrast level. Displayed are means and confidence intervals per condition

**a**

**b**

**c**

Association between errors

To investigate associations between the temporal and spatial error per trial, multilevel models with trial as first and participant as second level revealed a positive fixed effect of the spatial difference score on the temporal difference score [*χ*²(1) = 130.65, *p* < .001]. Additionally, there was a significant random effect of the spatial difference score [*χ*²(2) = 182.56, *p* < .001]. This means that, overall, the temporal difference score was higher when the spatial difference score was higher but there was significant variability between participants in this association.

Correlation with constant errors

There was a significant correlation between the temporal constant error and the time participants spend playing electronic games per day [*τ*= .32, *z* = 2.62, *p* = .008]. The more participants play electronic games, the later they touched the screen. Furthermore, there was a non-significant trend for a relationship between the temporal constant error and the time participants play electronic games on a touchscreen per day [*τ*= .22, *z* = 1.71, *p* = .086]. Again, the error increased with increasing amount of playing time. There were no other significant correlations [all *p*s > .253].

Correlation with variable errors

The correlation analysis revealed a significant negative association between the spatial variable error and hours of ball sport training per week [*τ*= -.32, *z* = -2.67, *p* = .007]. The more frequently participants played ball sports per week, the more precisely they hit the target. All other correlations did not reach significance [all *p*s > .201].

Comparison between both experiments

To examine whether the size of the constant and variable errors was similar across experiments, we compared the size of the error scores in the no-blur and highest contrast condition because those conditions were identical across experiments. Unpaired t-tests revealed no significant differences between experiments (all ps > .232). All error scores were quite similar across experiments.

General Discussion

Besides changes in blur or contrast, some other manipulations have been shown to impact interception performance in previous research as well as in the current study. Brenner et al. (2014) found that occluding the final part of a trajectory resulted in reduced temporal precision. During occlusion extrapolation is necessary to make any predictions of where and when a stimulus will be. Previous research has shown that extrapolation precision was decreasing with increasing distance to an occlusion point (Singh & Fulvio, 2005). In line with these findings, one would predict increasing spatial and temporal variability with increasing occlusion time. The current study supports this notion and thereby extends the findings of (Brenner et al., 2014) by showing that increasing the occluded temporal interval decreased not only temporal precision, but also spatial precision and spatial accuracy in both experiments (more undershooting, see Fig. 1b, 2b, 4b, 5b, 6b, and 8b in the OR1). Moreover, the temporal constant error decreased with increasing occlusion time, indicating that the longer the occlusion interval was, the earlier participants reacted (see Fig. 3b and 7b in the OR1). In line with research on time to arrival estimation (Benguigui et al., 2003), indicating that humans are unable to use information about acceleration (second-order information) and instead extrapolate constant velocities (frequently updating) one would expect less delayed responses for shorter occlusion times, because the later the ball is occluded the faster its latest visible velocity was. Supporting this idea, for the smallest occlusion time (where velocity is already very fast before occlusion) the temporal response is less delayed (see Fig. 3 and 7 in the OR1).

The temporal constraints induced by manipulating velocity should lead to increased temporal precision at the cost of decreased spatial precision. Similar to others (Tresilian et al., 2009), we only found a negative impact on the spatial precision (see Fig. 2a and 6a in the OR1), while the temporal variability was not significantly affected in either experiment (see Fig. 4a and 8a in the OR1). Yet, this is in conflict with others showing a significant decrease in the temporal variability (Lim, 2015) or even a significant increase in the spatiotemporal interception variability (Zhao & Warren, 2017). Interestingly, except for spatial precision also temporal accuracy decreased (earlier reactions) with increasing horizontal velocity (see Fig. 2a, 3a, 6a, and 7a in the OR1) which might be caused by faster interception movements found in previous research (e.g. Brouwer et al., 2000; Tresilian et al., 2003). In Experiment 1 the level of undershooting of the target trajectory decreased with increasing horizontal velocity (see Fig. 1a in the OR1), but this effect was not found in Experiment 2 (see Fig. 5a in the OR1).

In both experiments, the starting side had no impact on most of the error scores (see Fig. 1c-8c in the OR1). Only in Experiment 2 participants horizontally undershot targets slightly more, when they started at the right side, but this effect did not reach significance.

References

Benguigui, N., Ripoll, H., & Broderick, M. P. (2003). Time-to-contact estimation of accelerated stimuli is based on first-order information. *Journal of Experimental Psychology. Human Perception and Performance*, *29*(6), 1083–1101. https://doi.org/10.1037/0096-1523.29.6.1083

Bosco, G., Delle Monache, S., & Lacquaniti, F. (2012). Catching What We Can’t See: Manual Interception of Occluded Fly-Ball Trajectories. *PloS One*, *7*(11), e49381. https://doi.org/10.1371/journal.pone.0049381

Brenner, E., Driesen, B., & Smeets, J. B. J. (2014). Precise timing when hitting falling balls. *Frontiers in Human Neuroscience*, *8*, 342. https://doi.org/10.3389/fnhum.2014.00342

Brouwer, A.‑M., Brenner, E., & Smeets, J. B. J. (2000). Hitting moving objects. *Experimental Brain Research*, *133*(2), 242–248. https://doi.org/10.1007/s002210000371

Faul, F., Erdfelder, E., Buchner, A., & Lang, A.‑G. (2009). Statistical power analyses using G*Power 3.1: Tests for correlation and regression analyses. *Behavior Research Methods*, *41*(4), 1149–1160. https://doi.org/10.3758/BRM.41.4.1149

Faul, F., Erdfelder, E., Lang, A.‑G., & Buchner, A. (2007). G*Power 3: A flexible statistical power analysis program for the social, behavioral, and biomedical sciences. *Behavior Research Methods*, *39*(2), 175–191. https://doi.org/10.3758/BF03193146

Field, A., Miles, J., & Field, Z. (2013). *Discovering statistics using R*. Sage.

Lim, J. (2015). Effects of Spatial and Temporal Constraints on Interceptive Aiming Task Performance and Gaze Control. *Perceptual and Motor Skills*, *121*(2), 509–527. https://doi.org/10.2466/24.30.PMS.121c16x4

Singh, M., & Fulvio, J. M. (2005). Visual extrapolation of contour geometry. *Proceedings of the National Academy of Sciences of the United States of America*, *102*(3), 939–944. https://doi.org/10.1073/pnas.0408444102

Tresilian, J. R., Oliver, J., & Carroll, T. J. (2003). Temporal precision of interceptive action: Differential effects of target size and speed. *Experimental Brain Research*, *148*(4), 425–438. https://doi.org/10.1007/s00221-002-1309-0

Tresilian, J. R., Plooy, A. M., & Marinovic, W. (2009). Manual interception of moving targets in two dimensions: Performance and space-time accuracy. *Brain Research*, *1250*, 202–217. https://doi.org/10.1016/j.brainres.2008.11.001

Zhao, H., & Warren, W. H. (2017). Intercepting a moving target: On-line or model-based control? *Journal of Vision*, *17*(5), 12. https://doi.org/10.1167/17.5.12
